# C5aR1 signaling triggers lung immunopathology in COVID-19 through neutrophil extracellular traps

**DOI:** 10.1172/JCI163105

**Published:** 2023-06-15

**Authors:** Bruna M. Silva, Giovanni F. Gomes, Flavio P. Veras, Seppe Cambier, Gabriel V.L. Silva, Andreza U. Quadros, Diego B. Caetité, Daniele C. Nascimento, Camilla M. Silva, Juliana C. Silva, Samara Damasceno, Ayda H. Schneider, Fabio Beretta, Sabrina S. Batah, Icaro M.S. Castro, Isadora M. Paiva, Tamara Rodrigues, Ana Salina, Ronaldo Martins, Guilherme C.M. Cebinelli, Naira L. Bibo, Daniel M. Jorge, Helder I. Nakaya, Dario S. Zamboni, Luiz O. Leiria, Alexandre T. Fabro, José C. Alves-Filho, Eurico Arruda, Paulo Louzada-Junior, Renê D. Oliveira, Larissa D. Cunha, Pierre Van Mol, Lore Vanderbeke, Simon Feys, Els Wauters, Laura Brandolini, Andrea Aramini, Fernando Q. Cunha, Jörg Köhl, Marcello Allegretti, Diether Lambrechts, Joost Wauters, Paul Proost, Thiago M. Cunha

**Affiliations:** 1 Center for Research in Inflammatory Diseases (CRID), Department of Pharmacology, and; 2 Graduate Program in Basic and Applied Immunology, Ribeirão Preto Medical School, University of São Paulo, Ribeirão Preto, São Paulo, Brazil.; 3 Laboratory of Molecular Immunology, Department of Microbiology, Immunology and Transplantation, Rega Institute, KU Leuven, Leuven, Belgium.; 4 Department of Pathology and Legal Medicine, Ribeirão Preto Medical School, University of São Paulo, Ribeirão Preto, São Paulo, Brazil.; 5 Hospital Israelita Albert Einstein, São Paulo, Brazil.; 6 Department of Cell and Molecular Biology,; 7 Virology Research Center, and; 8 Divisions of Clinical Immunology, Emergency, Infectious Diseases and Intensive Care Unit, Ribeirão Preto Medical School, University of São Paulo, Ribeirão Preto, São Paulo, Brazil.; 9 Laboratory of Respiratory Diseases and Thoracic Surgery (BREATHE), Department of Chronic Diseases and Metabolism, and; 10 Laboratory for Clinical Infectious and Inflammatory Disorders, Department of Microbiology, Immunology and Transplantation, KU Leuven, Leuven, Belgium.; 11 R&D Department, Dompé Farmaceutici s.p.a., via Campo di Pile, L’Aquila, Italy.; 12 Division of Immunobiology, Cincinnati Children’s Hospital Medical Center, Cincinnati, Ohio, USA.; 13 Institute for Systemic Inflammation Research, University of Lübeck, Ratzebuger Allee, Lübeck, Germany.; 14 Laboratory of Translational Genetics, Department of Human Genetics, VIB-KU Leuven, Leuven, Belgium.; 15 Medical Intensive Care Unit, University Hospitals Leuven, Leuven, Belgium.

**Keywords:** COVID-19, Inflammation, Complement, Innate immunity, Molecular pathology

## Abstract

Patients with severe COVID-19 develop acute respiratory distress syndrome (ARDS) that may progress to cytokine storm syndrome, organ dysfunction, and death. Considering that complement component 5a (C5a), through its cellular receptor C5aR1, has potent proinflammatory actions and plays immunopathological roles in inflammatory diseases, we investigated whether the C5a/C5aR1 pathway could be involved in COVID-19 pathophysiology. C5a/C5aR1 signaling increased locally in the lung, especially in neutrophils of critically ill patients with COVID-19 compared with patients with influenza infection, as well as in the lung tissue of K18-hACE2 Tg mice (Tg mice) infected with SARS-CoV-2. Genetic and pharmacological inhibition of C5aR1 signaling ameliorated lung immunopathology in Tg-infected mice. Mechanistically, we found that C5aR1 signaling drives neutrophil extracellular traps-dependent (NETs-dependent) immunopathology. These data confirm the immunopathological role of C5a/C5aR1 signaling in COVID-19 and indicate that antagonists of C5aR1 could be useful for COVID-19 treatment.

## Graphical abstract

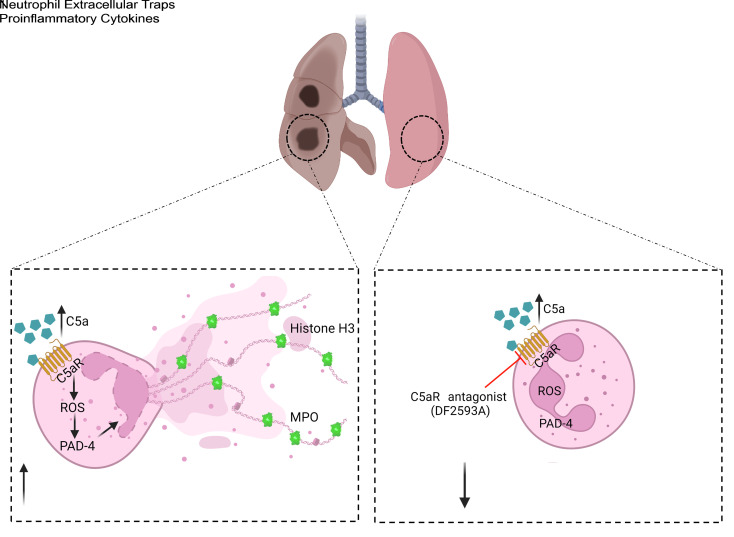

## Introduction

COVID-19 is the major acute global public health issue in this century. Patients with severe COVID-19 develop acute respiratory distress syndrome (ARDS), which may progress to organ dysfunction and death (1, 2). The disease itself is a consequence of infection with the SARS-CoV-2 virus, which triggers an inflammatory response by the host organism, potentially resulting in a maladaptive inflammatory response and progression to severe disease (3, 4). As in many other human viral diseases, pathology is mainly a consequence of the host’s response to the virus rather than the result of the virus itself. Therapy to reduce viral loads after development of the dysfunctional immune response may be considered as a therapeutic option but could be less favorable than appropriate control of inflammation. Combining antiviral therapy with immune control, including the development of specific antiinflammatory agents to block virus-triggered inflammatory responses, might be a strategic option to treat short-living virus-caused pathology, especially in COVID-19. This hypothesis has been confirmed by the demonstration that drugs targeting the inflammatory response are, at least in part, effective to control COVID-19 severity (5–10). Nevertheless, these therapies need to be used with caution, since they may also affect the host immune response against the virus and against secondary/opportunistic infections. Therefore, the development of novel agents to treat COVID-19 targeting the inflammatory/immune response should be focused on a mediator/process that is important for immune pathology but dispensable for infection control (11, 12). One possible candidate might be the complement component 5a/component 5a receptor type 1(C5a/C5aR1) signaling (11, 12).

C5a is one of the most important components of the complement cascade and possesses several proinflammatory actions (13, 14). C5a is a common component of the activation of all complement pathways and acts mainly via the G protein-coupled receptor (GPCR) C5aR1, also called CD88 (14). C5aR1 was initially identified in neutrophils, monocytes/macrophages, and mast cells (14, 15). The C5aR1 signaling has been implicated in the pathophysiology of several inflammatory diseases including virus-infection-induced diseases that cause lung pathology (16–19). For instance, C5a/C5aR1 inhibition alleviates lung damage in murine models of influenza A, Middle East respiratory syndrome coronavirus (MERS-CoV), and respiratory syncytial virus (RSV) (20–22).

A growing body of evidence suggests the possible participation of the complement system, and especially of C5a/C5aR1 signaling, in COVID-19 pathophysiology (23, 24). C5a levels increased in the blood of COVID-19 patients and correlated with disease severity (23). More recent clinical studies have shown a beneficial effect of anti-C5a therapies for COVID-19 (25–27), including a multicenter, double blind, randomized, placebo-controlled, phase III clinical trial (28). Nevertheless, no study performed an in-depth investigation of the outcome of the lack of or blockade of C5aR1 signaling on COVID-19 or the mechanisms behind its role. Herein, we found that C5a/C5aR1 signaling was increased in patients and in a preclinical mice model of COVID-19. Furthermore, we show that genetic and pharmacological blockage of C5aR1 signaling in myeloid cells (especially neutrophils) ameliorated COVID-19 lung immunopathology. Finally, we found that the C5aR1 signaling mediated COVID-19 immunopathology through enhancement of neutrophil extracellular traps (NETs) formation.

## Results

### C5a/C5aR1 signaling in the lung cells of patients with COVID-19.

**Figure 1:**
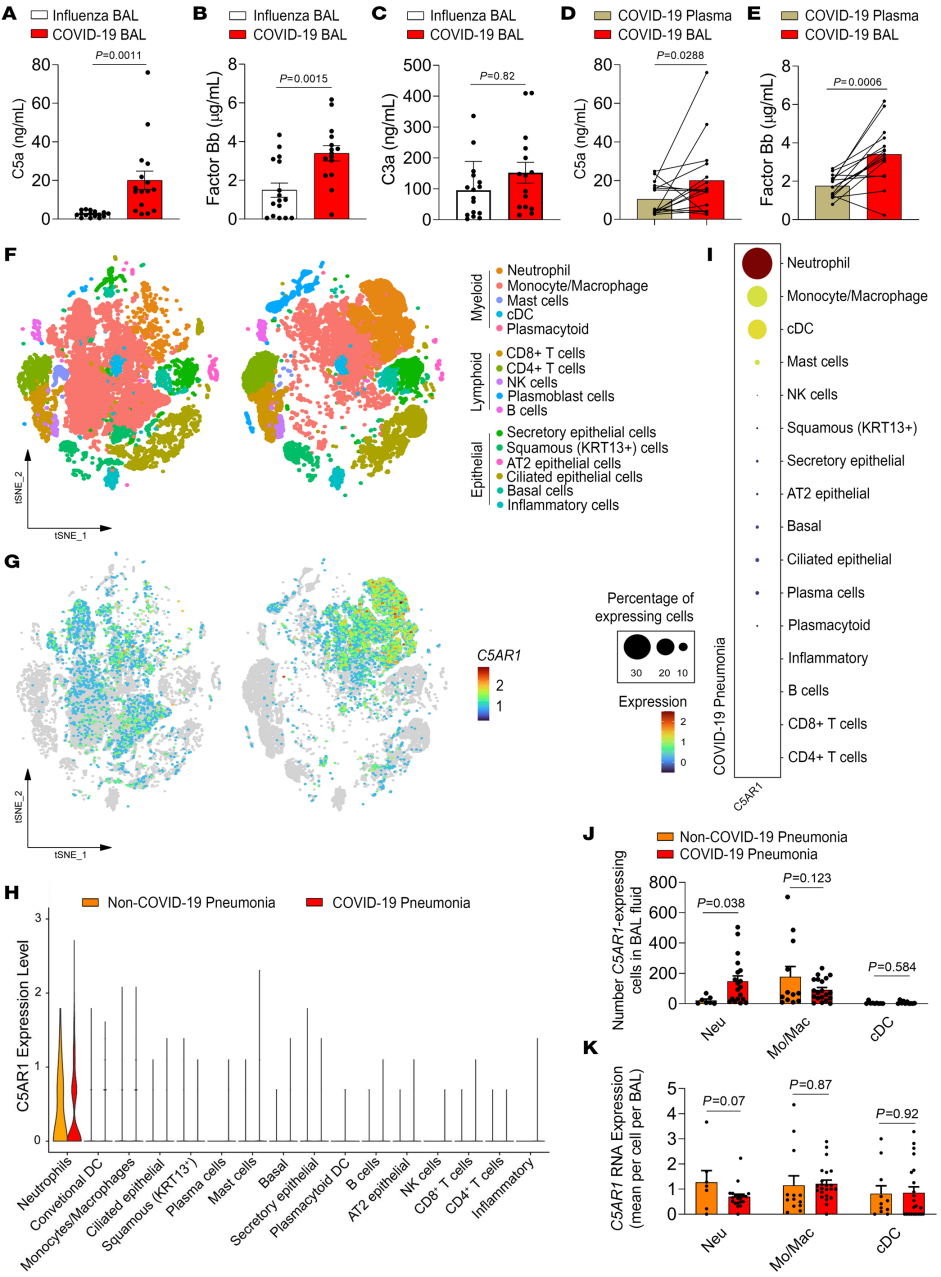
C5a levels and *C5AR1* expression in the BAL fluid and cells from patients with COVID- 19. An ELISA assay was performed to measure the concentrations of (**A**) C5a, (**B**) factor Bb, and (**C**) C3a in the BAL fluid from patients with influenza (*n* = 16) and patients with COVID- 19 (*n* = 16). (**B**) Paired concentrations of (**D**) C5a and (**E**) factor Bb in the plasma and BAL fluid from patients with COVID-19 were determined by ELISA. (**F**) A different cohort from a previously published data set was reanalyzed and the t-SNE analysis of total cells (65,166) from BAL fluid of patients with non-COVID-19 pneumonia (*n* = 13) and COVID-19 (*n* = 22) is shown. (**G**) Dot plots display the highlighted distribution of *C5AR1* for each indicated cell population. (**H**) Violin plots showing the expression levels of *C5aR1* in each type of cell from patients with COVID-19 or with non-COVID-19 pneumonia. (**I**) The dot plot depicts the scaled and centered expression of an average cell in each cluster and therefore contains negative and positive values. The average expression reflects the mean expression of *C5AR1* in each cluster compared with all other cells. (**J**) Number of cells per cell population [neutrophils (Neu), monocytes/macrophages (Mo/Mac), and dendritic cells (cDC)] that express *C5AR1* in the groups of patients with COVID-19 and non-COVID-19 pneumonia. (**K**) Average expression of *C5AR1* per cell for each cell population [neutrophils (Neu), monocytes/macrophages (Mo/Mac), and dendritic cells (cDC)] in the groups of patients with COVID-19 and non-COVID-19 pneumonia. Data are shown as the mean ± SEM. *P* values were determined by 2-tailed unpaired (**A**–**D**, **J**, and **K**) or paired (**D** and **E**) Student’s *t* tests followed by Wilcoxon matched-pairs signed rank tests.

In order to investigate the role of C5a/C5aR1 signaling in the pathophysiology of COVID-19, initially we assessed bronchoalveolar lavage (BAL) fluid from critically ill patients with COVID-19 requiring invasive mechanical ventilation. We previously reported that BAL fluid from these patients contained increased numbers of hyperactivated degranulating neutrophils and elevated concentrations of proinflammatory cytokines/chemokines (e.g., IL-1β, G-CSF, CXCL1, and CXCL8) compared with a non–COVID-19 viral pneumonia cohort of mechanically ventilated patients with influenza (29). We analyzed the levels of C5a in these cohorts of patient samples and found significantly higher C5a concentrations in the BAL fluid from patients with COVID-19 compared with influenza-infected patients (Figure 1A). Notably, the levels of factor Bb, but not of C3a, were higher in the BAL fluid from patients with COVID-19 compared with patients with influenza (Figure 1, B and C). In addition, the levels of C5a and factor Bb were higher in the BAL fluid compared with the corresponding paired plasma samples in patients with COVID-19 (Figure 1, D and E). Together, these results indicate that high C5a levels are produced locally (in lungs) in COVID-19, probably by the activation of alternative complement pathways, and correspond to stronger local-complement activation in COVID-19 compared with other severe viral lung infections.

The increased levels of C5a in the BAL fluid might indicate the activation of C5a-C5aR1 signaling. Thus, in an attempt to gain information about the possible role of C5a in the pathophysiology of COVID-19, we sought to identify the possible cell subtype in the BAL fluid of patients with COVID-19 expressing *C5AR1*, its main proinflammatory receptor (17, 30). To this end, we assessed our previously published database containing single-cell transcriptomes of BAL fluid cells from patients with COVID-19 and non-COVID-19 pneumonia and reanalyzed these data (31). We have found in our reanalyses (Figure 1F) that, among the different clusters of cells, in both groups, the expression of *C5AR1* was detected mainly in the neutrophil and monocyte/macrophage populations, and, to a limited extent, in conventional dendritic cells (cDC) (Figure 1, F–I). In addition, the number of *C5AR1-*expressing neutrophils was higher in the BAL fluid from patients with COVID-19 compared with BAL fluid from patients with non-COVID–19 pneumonia (Figure 1J). No differences were observed in the number of *C5AR1*–expressing monocytes/macrophages and cDC in these groups (Figure 1J). Notably, the average expression of *C5AR1* per cell of the BAL fluid is similar in both patients with COVID-19 and non-COVID-19 pneumonia (Figure 1K). The reanalyses of single-cell transcriptomics did not reveal the significant expression of C5 in the lung cells that was reported before (32) (Supplemental Figure 1; supplemental material available online with this article; https://doi.org/10.1172/JCI163105DS1), indicating that the increased levels of C5a could be mostly of hepatic origin.

A similar result related to the expression of *C5AR1* was revealed by the reanalyses of another public data set of the single-cell transcriptome of cells from BAL fluid of patients with COVID-19 (33), corroborating that *C5AR1*-expressing neutrophils are increased in the lungs of patients with COVID-19 (Supplemental Figure 2). Of note, this single-cell transcriptome data set also revealed some degree of expression of *C5AR1* in epithelial cells of the BAL fluid of COVID-19 patients (Supplemental Figure 2D).

**Figure 2:**
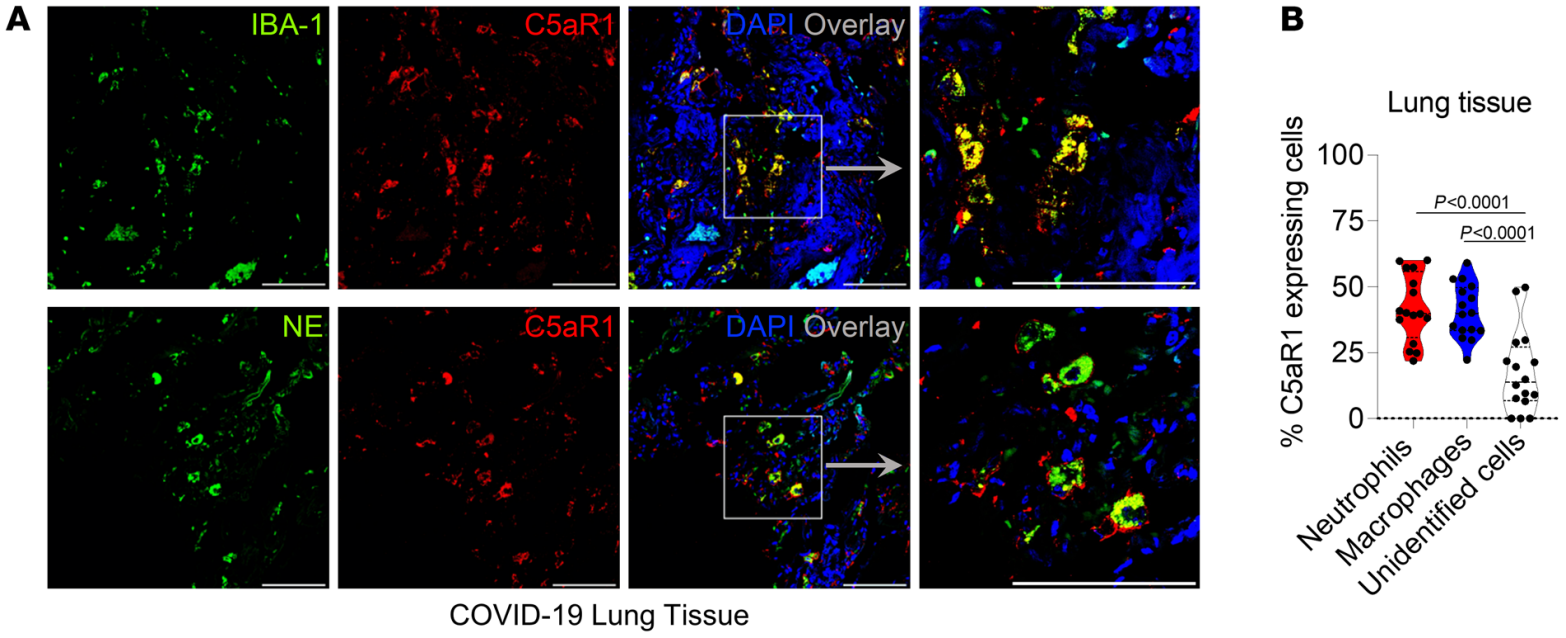
C5aR1 is expressed in macrophages and neutrophils in the lung tissue of patients with COVID-19. (**A**) Representative confocal images of the presence of C5aR1 in macrophages (Iba-1) and neutrophils (neutrophil elastase, NE) in the lung tissue from autopsies of patients with COVID-19 (*n* = 4 cases/4 randomized field). Cells were stained for nuclei (DAPI, blue), Iba-1, or NE (green), and C5aR1 (red). Scale bar: 50 μm. (**B**) Percentage of cells expressing C5aR1 in the COVID-19 lung. Data are shown as the mean ± SEM. *P* values were determined by 1-way ANOVA followed by Bonferroni’s posthoc test (**B**).

In order to validate the single-cell transcriptome data, lung tissue from post-mortem COVID-19 patients was used for C5aR1 immunostaining and costaining for neutrophil (neutrophil elastase; NE) and macrophage/monocyte (Iba-1) cellular markers. In agreement with the single-cell transcriptome, we found that C5aR1 is mainly expressed in NE^+^ cells (neutrophils; 41.87% ± 12.77%; Figure 1, I and J, and Supplemental Figure 4) and Iba-1^+^ cells (macrophage/monocytes, 40.87% ± 10.22%, Figure 2 and Supplemental Figure 3). The remaining nonidentified cells were 17.49% ± 15.52% (Figure 1B), which could be related to the epithelial cells that we found expressing *C5AR1* in the single-cell transcriptome analyses. Together, these data indicate that, in COVID-19, the enhanced production of C5a in the lung is mainly detected by neutrophils and/or macrophages/monocytes.

In an attempt to obtain further information about the possible role of C5a/C5aR1 signaling in the pathophysiology of COVID-19, we performed correlation analyses of C5a concentrations with different inflammatory markers/cells that we have previously shown to be enhanced in the BAL fluid of patients with COVID-19 (29). Notably, C5a levels correlated with the number of hyperactivated/degranulating neutrophils (positive for CD66b and the tetraspanin CD63) (Supplemental Figure 4) and with the neutrophil attractant CXCL8 but not with any other inflammatory marker (Supplemental Figure 4). In agreement, hyperactivated neutrophils in the BAL fluid of COVID-19 patients were characterized by higher expression of CXCL8 and they seem to play a critical role in COVID-19 pneumonia (29, 34–36). Altogether, these data point toward a possible role for C5a in the hyperactivation of neutrophils in the lungs of patients with COVID-19.

### C5a/C5aR1 signaling on myeloid cells has a detrimental role in a murine model of COVID-19.

**Figure 3:**
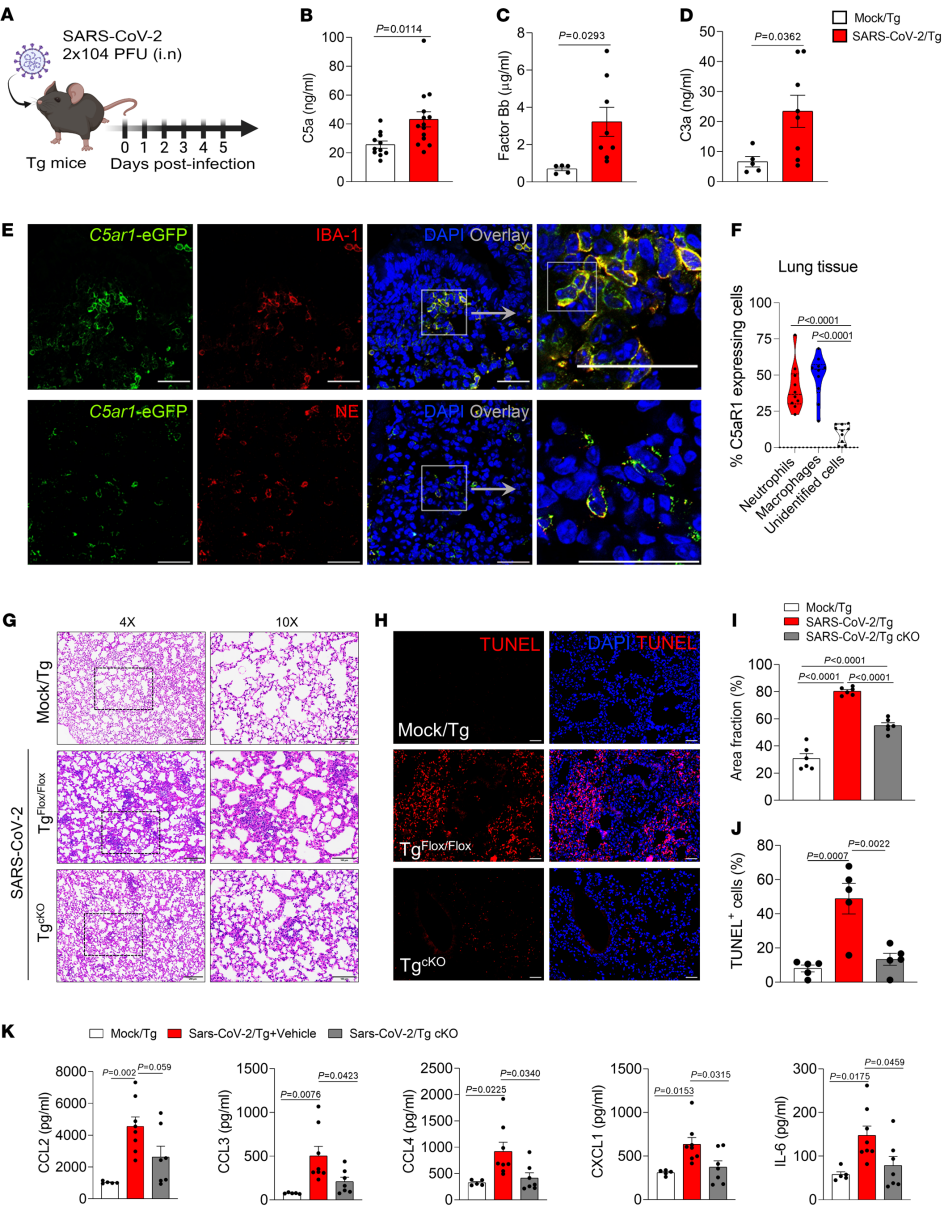
C5aR1 signaling on myeloid cells contributes to the lung pathology in a COVID-19 mouse model. (**A**) Tg mice were infected with SARS-CoV-2 (2 × 104 PFU, intranasally). ELISA assay to measure levels of (**B**) C5a in the lung homogenate of infected animals (*n* = 14) or mock control (*n* = 11). (**C**) factor Bb and (**D**) C3a levels in the lung homogenate of infected animals (*n* = 8) or mock control (*n* = 5). (**E**) Representative confocal images of the presence of C5aR1 expression in the lung tissue of Tg^fl/fl^ mice (*C5ar1*-eGFP mice) infected with SARS-CoV-2 (5 dpi). Tissue slices were costained for nuclei (DAPI, blue), Iba-1 (macrophages, red) and NE (neutrophils, red) markers. Scale bar: 50 μm. (**F**) Percentage of cells expressing C5aR1 in the lung tissue of Tg^fl/fl^ mice infected with SARS-CoV-2 (*n* = 4 mice/4 randomized field). (**G**) Representative H&E staining from the lung of SARS-CoV-2-infected Tg^fl/fl^(*n* = 6) or Tg^cKO^ mice (*n* = 6). A mock-infected group was used as control (*n* = 6). Scale bars: 200 μm (4 ×), 100 μm(10 ×). (**H**) TUNEL staining (red) for detection of apoptotic cells in situ from lung tissue of SARS-CoV-2–infected Tg^fl/fl^ (*n* = 5) or Tg^cKO^ mice (*n* = 6). Mock-infected Tg mice were used as a control (*n* = 5/group). (**I**) Quantification of the lung septal area fraction. (**J**) Percentage of TUNEL positive cells in lung tissue. Scale bar: 50 μm. (**K**) ELISA assays were performed to detect CCL2, CCL3, CCL4, CXCL1, and IL-6 levels in the lung tissue of Tg^fl/f^ (*n* = 8) or Infected Tg^cKO^ mice (*n* = 7). Mock-infected Tg mice were used as a control (*n* = 5). Data are shown as the mean ± SEM. *P* values were determined by (**B**–**D**) Student’s 2-tailed *t* test and (**F**, **I**, **J**, and **K**) 1-way ANOVA followed by Bonferroni’s posthoc test.

In order to better understand the importance and role of C5a/C5aR1 signaling on the pathophysiology of COVID-19, we moved to a well-established preclinical mouse model used to study this disease, the K18-hACE2 Tg mice (Tg mice) infected with SARS-CoV-2 (37, 38) (Figure 3A). As observed in BAL fluid from patients with COVID-19, the levels of C5a increased in the lungs of Tg mice infected with SARS-CoV-2 (Figure 3B). We also detected increased levels of factor Bb and C3a in the lungs of SARS-CoV-2–infected Tg mice (Figure 3, C and D).

We also noticed that clinical signals (clinical score and weight loss), lung dysfunction (reduction of oxygen saturation), and lung pathology (focal area of neutrophil infiltration into the alveolar space, type II alveolar epithelial cell proliferation, focal filling of the alveolar space with proteinaceous alveolar fluid and debris, and thickening of alveolar septae by inflammatory cells) worsened in the COVID-19 mouse model compared with noninfected mice (Supplemental Figure 5, A and B, and Supplemental Figure 6). These observations were associated with increased levels of proinflammatory cytokines/chemokines in the lungs of infected mice (Supplemental Figure 5C), as observed previously (37, 39, 40). The expression of C5aR1 in lung tissue of SARS-CoV-2–infected mice was also analyzed by immunofluorescence. Tg*^fl/fl^* mice (which contain an eGFP reporter for C5aR1 expression) were infected with SARS-CoV-2 and the lungs were collected at 5 days postinfection (dpi). Similar to what we observed in the lung tissue of COVID-19 patients, immunofluorescence analyses of the lung tissue of SARS-CoV-2-infected Tg*^fl/fl^* mice revealed that C5aR1 was mainly expressed in cells positive for NE (neutrophils, 41.2% ± 16.07%) and Iba-1 (macrophages, 48.62% ± 15.07%) (Figure 3, E and F). The C5aR1 seemed to be expressed by 10.17% ± 6.08% of unidentified cells (Figure 3F). These results indicate that, during SARS-CoV-2 infection in mice, there may also be a local activation of C5a/C5aR1 signaling, especially in neutrophils and macrophages/monocytes.

Based on the fact that the pattern of expression of C5aR1 was mainly concentrated in myeloid cells (neutrophils and macrophages/monocytes) in the lung of patients with COVID-19 and SARS-CoV-2–infected Tg mice, we developed a colony of Tg mice lacking C5aR1 signaling (Tg^cKO^ mice) in these immune cells and infected them with SARS-CoV-2 (Supplemental Figure 7A). Although we did not observe any difference in the weight loss or clinical score in Infected Tg^cKO^ mice compared with Tg^fl/fl^ mice during the course of the disease (Supplemental Figure 7B), the histopathological analysis of the lung revealed a reduced level of tissue damage in Tg^cKO^ mice (Figure 3, G and I, and Supplemental Figure 6). In agreement with the histopathological data, the number of TUNEL-positive cells in the lung tissue of Tg^cKO^ mice was also reduced when compared with the tissue of Tg^fl/fl^ mice, indicating a reduction in cell death and, consequently, a reduction in the lung tissue damage (Figure 3, H and J). We performed ELISA assays to the cytokines that we noticed altered in the mouse model (Supplemental Figure 5C) and observed that the reduction in COVID-19–related lung pathology in Infected Tg^cKO^ mice was also associated with a reduction in the levels of proinflammatory cytokines/chemokines, especially, CCL3, CCL4, CXCL1, and IL-6 (Figure 3K). No difference was observed in the viral load between Tg^fl/fl^ and infected Tg^cKO^ mice (Supplemental Figure 7C). These results indicated that C5aR1 signaling on myeloid cells was involved in the SARS-CoV-2–induced lung pathology but had no participation in the control of the virus infection.

### A pharmacological C5aR1 antagonist ameliorates COVID-19 in the mouse model.

**Figure 4:**
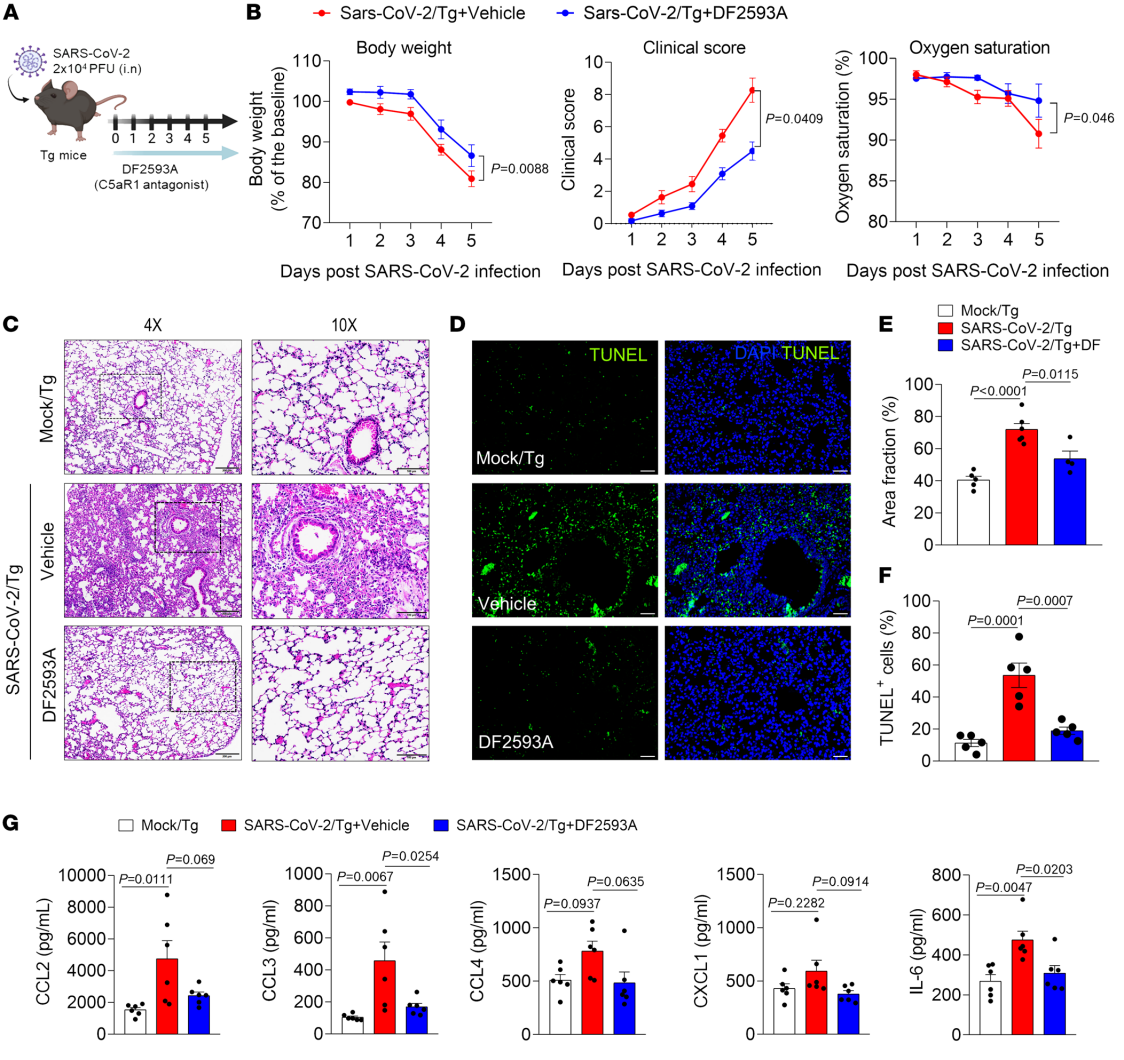
DF2593A, a selective C5aR1 antagonist, ameliorates COVID-19 in mice model. (**A**) Tg mice were infected with SARS-CoV-2 (2 x 104 PFU, intranasally) and treated with DF2593A (3 mg/kg, p.o) 1 hour before SARS-CoV-2 infection and once a day up to the day of sample collection (5 dpi). (**B**) Body weight, clinical score, and oxygen saturation were measured daily after infection (*n* = 11/group, pooled from 2 independent experiments). (**C**) Representative H&E staining from the harvested lung of the COVID-19 mouse model treated (*n* = 4) or not (*n* = 6) with DF2593A. A mock-infected group was used as control (*n* = 5). Scale bars: 200 μm (4 ×); 100 μm (10 ×). (**D**) TUNEL staining (green) for detection of apoptotic cells in situ from lung tissue of mice (*n* = 5/group). (**E**) Quantification of the lung septal area fraction. (**F**) Percentage of TUNEL-positive cells in lung tissue. Scale bar: 50 μm. (**G**) ELISA assays were performed to detect CCL2, CCL3, CCL4, CXCL1, and IL-6 levels in lung homogenate (*n* = 6/group). A mock-infected group was used as the control group. Data are shown as the mean ± SEM. *P* values were determined by 1-way ANOVA followed by Bonferroni’s posthoc test (**E**–**G**).

Since C5a/C5aR1 signaling seems to be involved in the immunopathology of COVID-19, we sought to test the efficacy of DF2593A, an orally acting and selective C5aR1 allosteric antagonist (41), on SARS-CoV-2–infected Tg mice to explore this candidate for the treatment of COVID-19. As a proof-of-concept experiment, we treated Tg mice with DF2593A 1 hour before SARS-CoV-2 infection and once a day up to the day of sample collection (5 dpi) (Figure 4A). Notably, the treatment with DF2593A reduced the body weight loss, improved the clinical score, and mitigated the reduction of oxygen saturation (Figure 4B) of the Tg-infected mice compared with vehicle-treated mice. This treatment also ameliorated lung pathology and reduced the number of dead cells (TUNEL+ cells) in the lung tissue of DF2593A-treated Tg-infected mice when compared with the vehicle-treated group (Figure 4, C–F, and Supplemental Figure 6), while it did not alter the viral load (Supplemental Figure 8A). Corroborating these results, in vitro data showed that DF2593A was also not effective to inhibit SARS-CoV-2 replication in Vero E6 cells (Supplemental Figure 8B). We performed ELISA assays to the cytokines that we noticed altered in the mouse model (Supplemental Figure 5C) and we observed that the reduction in lung pathology was also associated with a reduction in the levels of proinflammatory cytokines/chemokines, especially CCL3 and IL-6, in the lung tissue of mice treated with DF2593A (Figure 4G).

**Figure 5:**
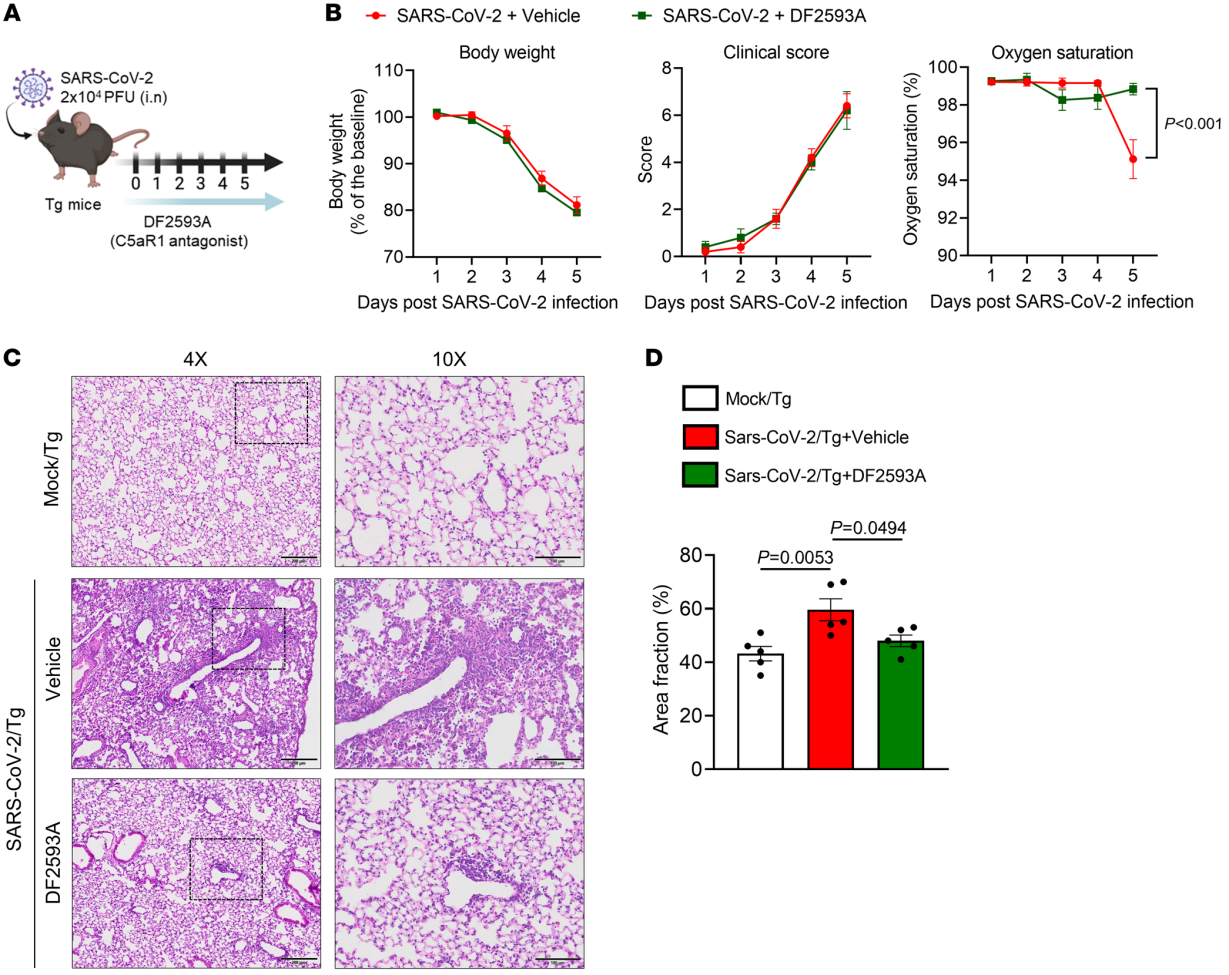
The postinfection treatment with DF2593A reduced lung pathology/disfunction in SARS-CoV-2-infected Tg mice. (**A**) Tg mice were infected with SARS-CoV-2 (2 × 10^4^ PFU, intranasally) and treated with DF2593A (3 mg/kg, p.o) 24 hours after SARS-CoV-2 infection and once a day up to the day of sample collection (5 dpi). (**B**) Body weight, clinical score, and oxygen saturation were measured daily after infection (*n* = 5/group). (**C**) Representative H&E staining from the harvested lung of the COVID-19 mouse model treated or not with DF2593A (*n* = 5/group). A mock-infected group was used as control (*n* = 5). Scale bars: 200 μm (4 ×); 100 μm (10 ×). (**D**) Quantification of the lung septal area fraction. Data are shown as the mean ± SEM. *P* values were determined by 1-way ANOVA followed by Bonferroni’s posthoc test.

In a therapeutic perspective, we performed a postinfection treatment (starting 24 hours after infection) of infected mice with DF2593A (Figure 5A). Although, we did not find a significant difference in the clinical evolution of the disease and loss of body weight, the DF2593A postinfection mitigated the reduction of oxygen saturation (Figure 5B) and lung pathology (Figure 5, C and D, and Supplemental Figure 6) when compared with infected Tg mice treated with vehicle. These preclinical results indicate that pharmacological inhibition of C5aR1 could be a novel approach to ameliorate COVID-19.

### C5a/C5aR1 signaling enhances NETs formation to aggravate COVID-19.

C5a/C5aR1 signaling in myeloid cells (especially in neutrophils) is able to promote cell migration by triggering their arrest on the endothelium and/or chemotaxis (17, 42), suggesting that it would be involved in the recruitment of these cells into the SARS-CoV-2 infected lungs. Thus, we further analyzed whether the lack of C5aR1 signaling in myeloid cells could impact the infiltration of these cells in the lung of SARS-CoV-2–infected Tg mice. Notably, FACS analyses revealed that the infiltration of total leukocytes (CD45^+^ cells), myeloid cells (CD45^+^CD11b^+^) as well as neutrophils (CD45^+^CD11b^+^Ly6G^+^ cells) and inflammatory monocytes (CD11b^+^CCR2^+^Ly6C^+^) was similar in the lung tissue of infected Tg^cKO^ mice compared with Tg^fl/fl^ mice (Supplemental Figure 9, A–D). Similar to what we have found in Tg^cKO^ mice, DF2593A treatment did not reduce the infiltration of total myeloid cells, neutrophils, or inflammatory monocytes (Supplemental Figure 9, E–G) in the lung tissue of Tg-infected mice. On the other hand, the total leukocyte infiltration in the lung tissue of Tg-infected mice was reduced by DF2593A treatment compared with vehicle treatment (Supplemental Figure 9H). Together, these results indicated that C5aR1 signaling on myeloid cells was not crucial in the infiltration of these cells into the lung of SARS-CoV-2–infected Tg mice.

**Figure 6:**
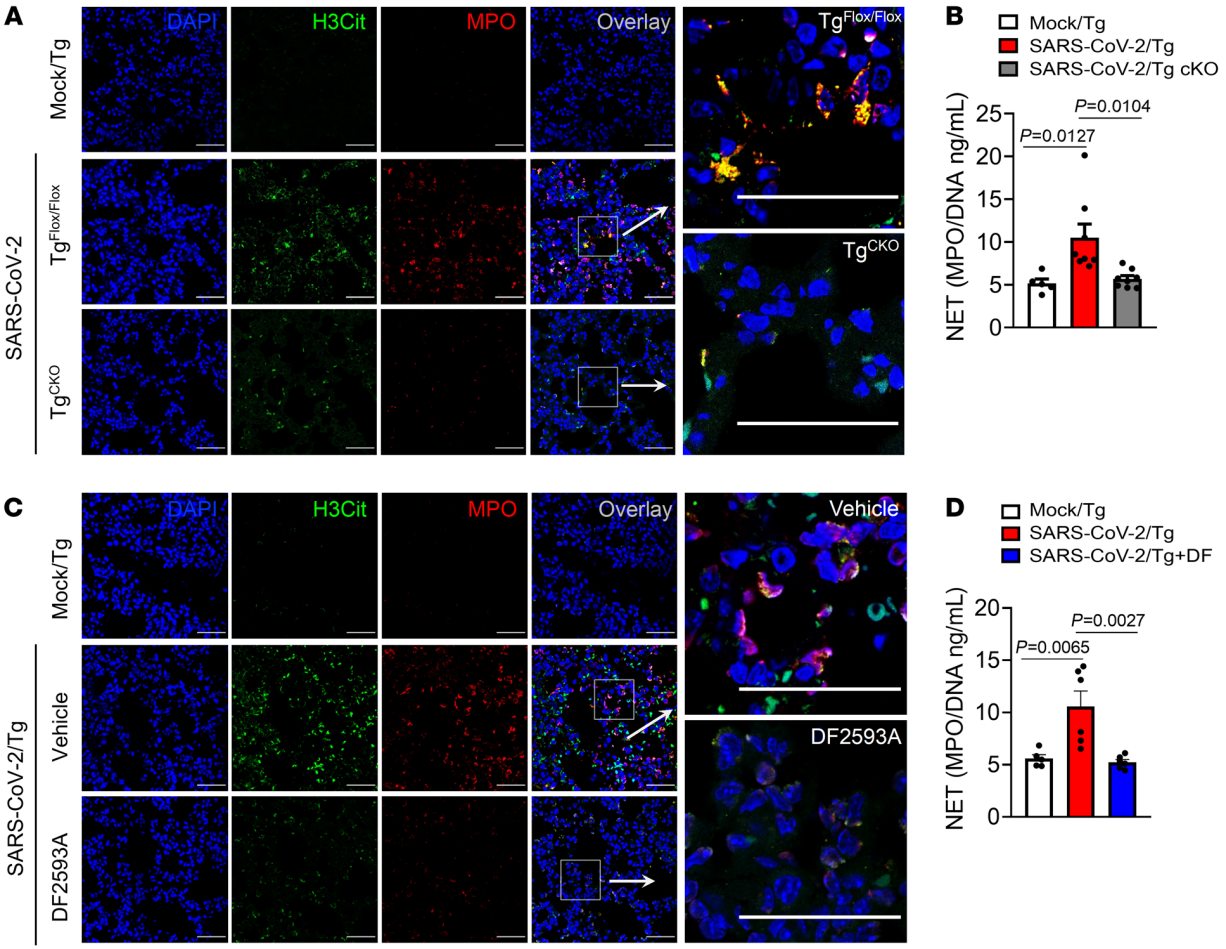
C5a/C5aR1 signaling is involved in the pathophysiology of COVID-19 through NET formation. Tg^fl/fl^ (*n* = 8) and Tg^cKO^ (*n* = 8) mice were infected with SARS-CoV-2 (2 × 10^4^ PFU, intranasally). (**A**) Representative confocal images showing the presence of NETs in the lung tissue from Tg^fl/fl^ or Infected Tg^cKO^ mice. A mock- infected group was performed as control (*n* = 5). Staining shows nuclei (DAPI, blue), H3Cit (green), and myeloperoxidase (MPO) (red). (**B**) At 5 dpi, the levels of NETs were quantified by MPO-DNA PicoGreen assay in the supernatant of the lung homogenate. (**C**) Tg-infected mice were treated with DF2593A (3mg/kg, p.o, *n* = 6) or vehicle (*n* = 5/group). Representative confocal images showing the presence of NETs in the lung tissue of Tg-infected mice treated with DF2593A or vehicle (*n* = 5/group). A mock-infected group was performed as control (*n* = 5). (**D**) At 5 dpi, NETs levels were quantified by MPO-DNA PicoGreen assay in the supernatant of the lung homogenate. Data are shown as the mean ± SeM. *P* values were determined by 1-way ANOVA followed by Bonferroni’s posthoc test (**B** and **D**). Scale bar: 50 μm.

Our findings indicating that C5a/C5aR1 signaling in myeloid cells was involved in the lung immunopathology of COVID-19, but not in the infiltration of these cells into the lung, prompted us to hypothesize that this signaling would be involved in the local activation of these cells. Additionally, our finding that C5a levels in the BAL of COVID-19 patients correlated with degranulation of hyperactivated neutrophils and proinflammatory cytokines/chemokines (Supplemental Figure 4) also supported this hypothesis. Among the downstream mechanisms by which activated neutrophils might participate in the pathophysiology of COVID-19, the production of NETs is one of the most described (43, 44). In our lung tissue samples from COVID-19 patients, we also detected the presence of NETs (Supplemental Figure 10). Thus, we evaluated whether C5a/C5aR1 signaling would be involved in NETs formation in the lungs of SARS-CoV-2–infected Tg mice. Corroborating this hypothesis, we found that the levels of NETs in the lung tissue of infected Tg^cKO^ mice were significantly reduced compared with the Tg^fl/fl^-infected mice (Figure 6, A and B). Furthermore, we found that the lung tissue of Tg-infected mice treated with DF2593A has lower levels of NETs compared with the lung tissue from vehicle-treated mice (Figure 6, C and D).

**Figure 7:**
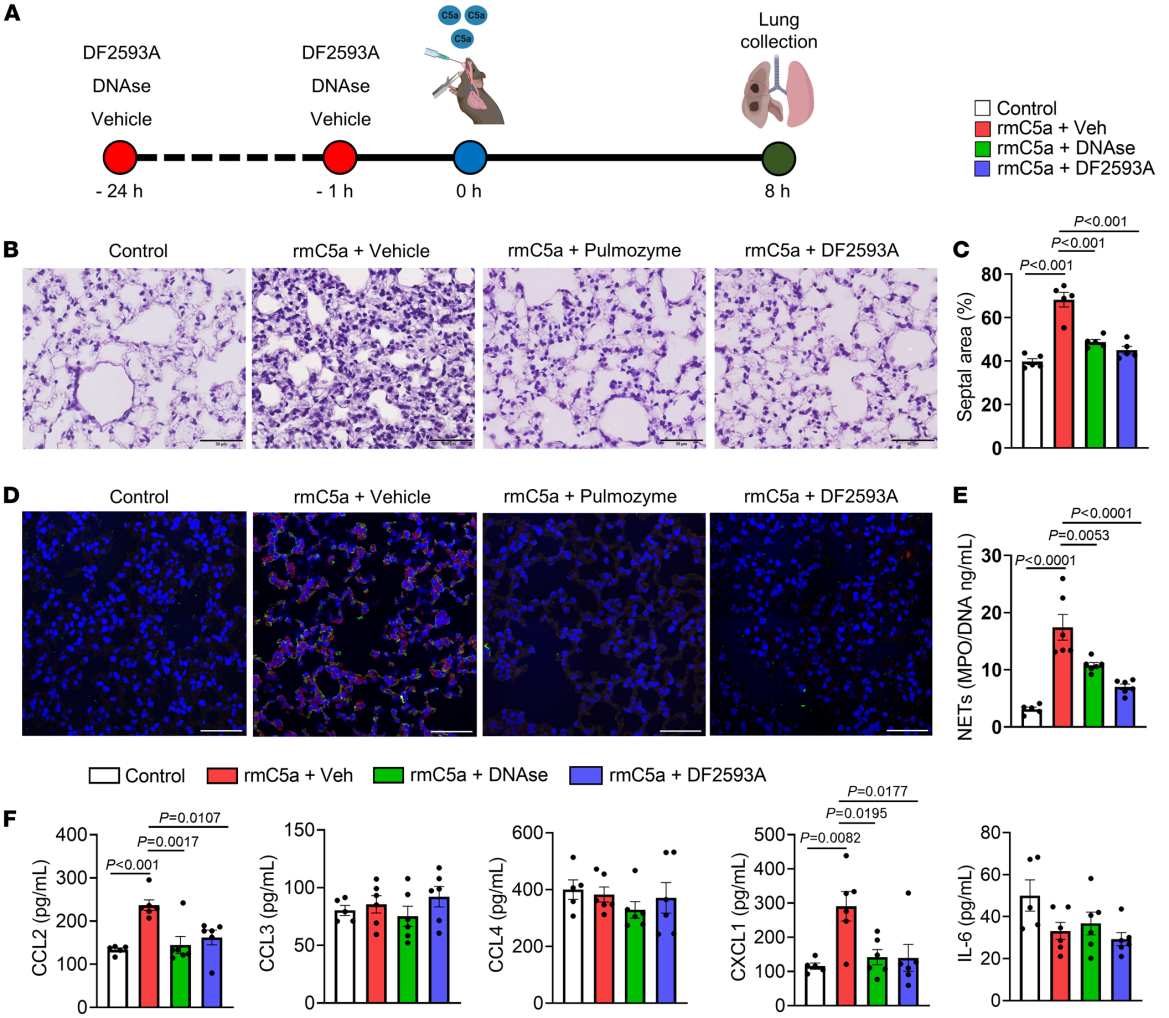
Intratracheal instillation with C5a induced lung immunopathology via C5aR1 signaling and NETs. (**A**) C57/BL6 mice were treated twice with vehicle, DNAse (Pulmozyme, 10 mg/kg, s.c.), or C5aR1 antagonist (DF2593A, 3 mg/kg, orally), 24 hours and 1 hour before the intratracheal instillation of rmC5a (400 ng). (**B**) Lung slices from the control group or mice challenged with rmC5a and treated with vehicle, DNAse, or C5aR1 antagonist (DF2593A) were stained with H&E for evaluation of histological changes. (**C**) Quantification of the lung septal area fraction (*n* = 5/group). (**D**) Lung slices from the control group or from mice challenged with rmC5a and treated with vehicle, DNAse, or C5aR1 antagonist (DF2593A) were costained for nuclei (DAPI, blue), H3Cit (green), and MPO (red) markers. (**E**) NET quantification by the MPO-DNA PicoGreen assay in the supernatant of the lung homogenate (*n* = 5–6/group). (**F**) ELISA assays were performed to detect CCL2, CCL3, CCL4, CXCL1, and IL-6 levels in lung homogenate (*n* = 5–6/group). Data are shown as the mean ± SEM. *P* values were determined by 1-way ANOVA followed by Bonferroni’s posthoc test (**C**, **E**, and **F**).

Instillation of C5a in the mouse lungs has been shown to promote tissue inflammation and damage (45). To test that the deleterious effects of C5a described above are dependent on NETs in vivo, we treated C57BL6 mice twice with DNAse [NETs degrading agent (46)] or DF2593A following the intratracheal instillation of recombinant murine (rm) C5a (Figure 7A). Intratracheal instillation of rmC5a promoted lung pathology associated with the presence of NETs and increased levels of CCL2 and CXCL1 (Figure 7, B–F). Both treatments (DNAse and DF2593A) reduced these alterations induced by mrC5a installation to the levels found in control animals (Figure 7, B–F). These results indicated that C5a-induced lung inflammation/pathology was dependent on NETs release through C5aR1 signaling.

**Figure 8:**
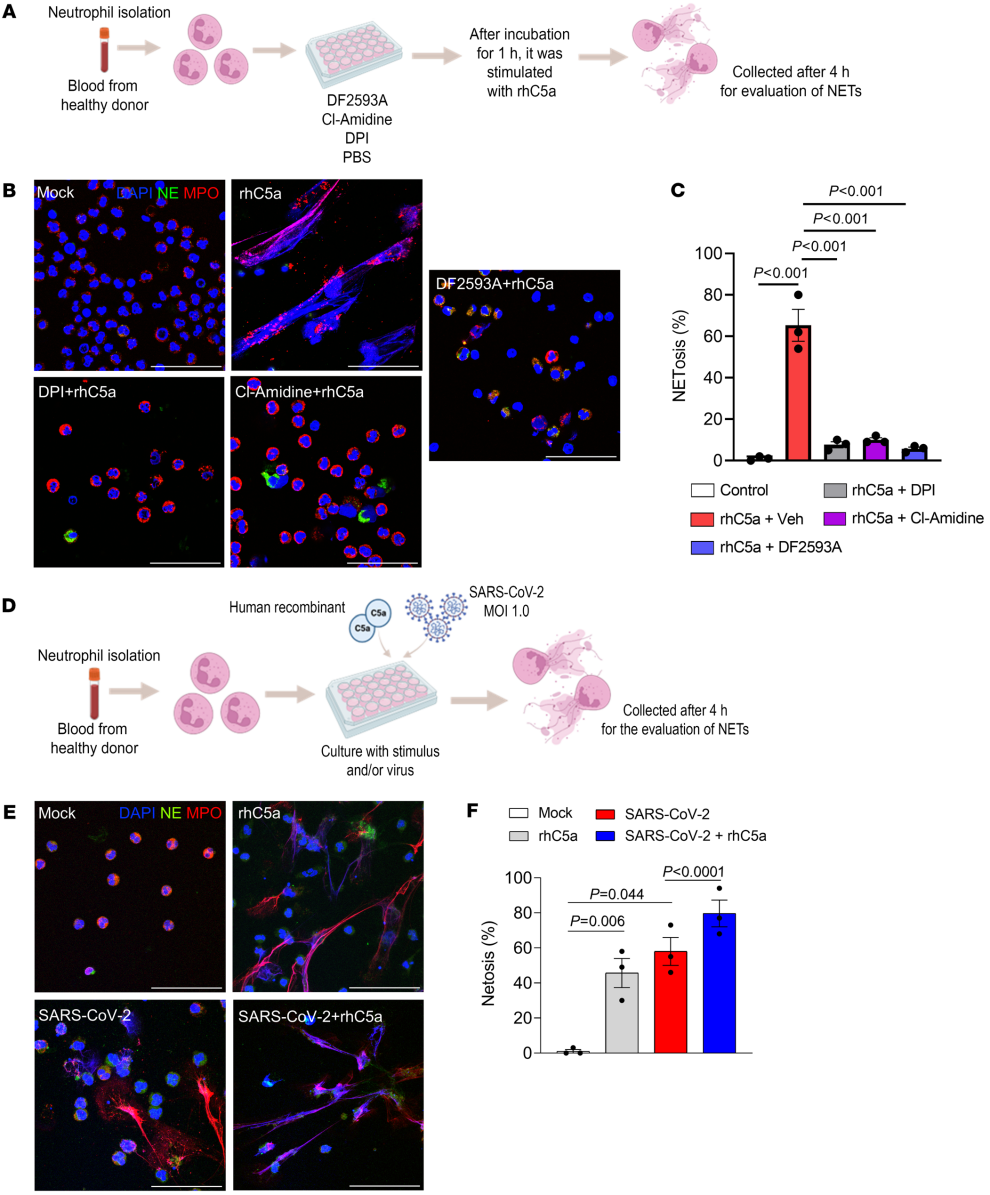
C5a is able to directly promote and enhance SARS-CoV-2-induced NETosis. (**A**) Isolated human neutrophils were incubated with PBS, DPI, Cl-amidine, or DF2593A for 1 h and then challenged with rhC5a (3 nM) for 4 h. (**B**) Cells were stained for nuclei (DAPI, blue), NE (green), and MPO (red). (**C**) Percentage of NETs quantification in these neutrophils supernatants (*n* = 3 donors). (**D**) Neutrophils were isolated from healthy donors and incubated with mock, rhC5a (3 nM), and SARS-CoV-2 (MOI = 1.0) for 4 hours. One group of SARS-CoV-2-infected cells was pretreated with rhC5a (3 nM). (**E**) Representative images of NETs release. Cells were stained for nuclei (DAPI, blue), NE (green), and MPO (red). Scale bar: 50 μm. (**F**) Percentage of NETs quantification in these neutrophils supernatants (*n* = 3 donors). Data are shown as the mean ± SEM. *P* values were determined by 1-way ANOVA followed by Bonferroni’s posthoc test (**C** and **F**).

The importance of NETs for the proinflammatory action of C5a/C5aR1 signaling in these models, described above, could be due to a direct or indirect effect on neutrophils. In this context, we evaluated the ability of C5a to induce NETs in an in vitro culture of human blood–derived neutrophils. Notably, we found that the treatment of human neutrophils with recombinant human (rh) C5a promoted NETosis (Figure 8, A–C). Mechanistically, we found that rhC5a-induced NETosis was inhibited by the treatment of human neutrophils with DF2593A, CL-amidine (PAD4 inhibitor), and diphenyleneiodonium (DPI; Reactive oxygen species, ROS inhibitor) (Figure 8, A–C). In addition, neutrophils infected with SARS-CoV-2 produced higher levels of NETs in the presence of low concentration of rhC5a when compared with rhC5a-treated neutrophils or infected neutrophils without addition of rhC5a (Figure 8, D–F). These results suggest that C5a via C5aR1 is able to directly promote NETosis through the stimulation of the canonical PAD4-ROS pathway. The data also indicate that in the SARS-CoV-2 infecting neutrophils, C5a/C5aR1 signaling might amplify the NETosis process. Altogether, these data indicate that the induction of NETs in the lung tissue of SARS-CoV-2–infected mice might be a crucial mechanism triggered by C5a/C5aR1 signaling that contributes to the pathophysiology of COVID-19.

## Discussion

COVID-19 is caused by 2 main factors: the virus replication that causes cellular injury and the dysregulated inflammatory/immune response that amplifies the tissue/organ dysfunction, especially in the lung. Although there is a race to identify novel antiviral drugs capable to inhibit SARS-CoV-2 replication and reduce COVID-19 severity, drugs that target the inflammatory/immune response, at least partially, have been shown effective in ameliorating COVID-19 (47–51). With regard to drugs targeting the immune system to control COVID-19, it is desirable to identify immune cells/mediators and molecular mechanisms that are not involved in the control of viral infection (and possible secondary infection) but are critical for immunopathology. Among several inflammatory mediators that may possess these characteristics, we and others consider complement factor C5a and its receptor, C5aR1, among the most interesting candidates (11, 30). Targeting C5a/C5aR1 signaling ameliorates virus infection–induced lung diseases, including influenza A, MERS-CoV, and RSV (20–22). Herein, we confirmed this hypothesis showing that both genetic and pharmacological inhibition of C5a/C5aR1 signaling, especially in neutrophils, had a beneficial effect on a preclinical mouse model of COVID-19. In addition, we showed that this beneficial effect was likely due to a reduction in NETs formation.

The understanding of COVID-19 pathophysiology is one of the most important ways to identify critical targets for the development of novel drugs to treat this disease. In this context, our study provides evidence validating the hypothesis that C5a/C5aR1 signaling plays a detrimental role to patients with COVID-19 and might be considered as an interesting candidate for novel treatments. Initially, we showed that C5aR1 signaling is selectively enhanced in the lungs of patients with COVID-19 compared with patients with the influenza virus, especially in neutrophils. These data are in agreement with previous reports showing higher levels of C5a in the plasma of patients with COVID-19, which correlate with disease severity (24, 52–54). Our data on the increase of factor Bb in the BAL fluid from patients with COVID-19 are also consistent with the observation of systemic activation of the alternative complement pathway (55–57). In addition, our human data were validated in a well-accepted preclinical model of COVID-19, in which we also observed an increase in C5aR1 signaling activation in myeloid cells (especially neutrophils) in the lung after SARS-CoV-2 infection.

The increase in C5aR1 signaling in the lungs of patients and mice with COVID-19 led us to explore whether inhibition of this pathway would have a protective effect. It should be noted that mice lacking C5aR1 signaling in myeloid cells (Tg^cKO^ mice) and use of the pharmacological inhibitor (the C5aR1 antagonist, DF2593A) provided beneficial effects. The dissociation between clinical parameters and lung pathology in infected Tg^cKO^ mice might be explained by the fact that, while Tg mice infected with SARS-CoV-2 developed lung disease similar to patients with COVID-19, clinical signs that led to eventual morbidity/mortality are mainly due to CNS dysfunction (58, 59). In fact, high SARS-CoV-2 burden and encephalitis have also been found in the brains of these animals (58–60). This has been considered a limitation of this COVID-19 mouse model (58). Alternatively, we cannot exclude that C5aR1 signaling in cell types, beyond neutrophils/macrophages, might also play a role in the pathophysiology of COVID-19 (61, 62). For instance, C5aR1 signaling in endothelial cells was found to be a prothrombogenic effector in COVID-19 patients (62). Thus, further studies will be necessary to address the role of C5a/C5aR1 signaling in cells other than myeloid cells in the pathophysiology of COVID-19. In addition, the higher efficacy of DF2593A on clinical parameters in Tg mice infected with SARS-CoV-2 compared with the phenotype observed in Tg^cKO^ mice was not immediately apparent, but it could be also explained by the fact that C5aR1 is expressed in cells other than myeloid cells, which are probably inhibited by the C5aR1 antagonist as well. Additionally, since we had previously shown that DF2593A was able to cross the blood-brain barriers (41), it might also reduce brain inflammation, which is an important drawback of this COVID-19 mouse model. Nevertheless, since CNS changes have been considered one of the important aspects of Long-COVID-19 (63) syndrome, the blockage of C5aR1 signaling by DF2593A could be an alternative to avoid the development of this condition. Supporting this hypothesis, in a mouse model of MERS-CoV infection, brain damage was reduced by an anti-C5aR1 murine antibody (64).

Regarding the mechanisms by which C5aR1 signaling is involved in the lung immunopathology during COVID-19, we ruled out the possibility that this pathway would be crucial in the recruitment of myeloid cells into the SARS-CoV-2-infected lungs. Indeed, no significant alteration in myeloid cell infiltration in the lungs of COVID-19 mice was observed either with genetic or pharmacological inhibition of C5aR1 signaling. This could be due the redundancy among the different inflammatory mediators such as neutrophil/monocytes–recruiting chemokines (e.g., CXCR2 ligands and CCL2), which are upregulated in the lungs of SARS-CoV-2-infected mice (38, 64). On the other hand, the inhibition of C5aR1 by DF2593A in cells beyond myeloid cells might explain the reduction of total leukocyte infiltration caused by pharmacological treatment. Indeed, C5aR1 signaling on nonmyeloid cells might favor, directly or indirectly, the infiltration of nonmyeloid leukocytes during COVID-19 infection in mice. Moreover, these nonmyeloid cells (e.g., NK cells) can be harmful to the lungs during COVID-19, as has been demonstrated (65). This might also explain why we noticed an improvement in overall clinical signs in mice treated with DF2593A, but not in the model of mice lacking C5aR1 in myeloid cells (Tg^cKO^).

Since C5a/C5aR1 signaling in myeloid cells is involved in the lung immunopathology of COVID-19 but not in the infiltration of these cells into the lung, we investigated its possible role in the local activation of these cells, focusing mainly on neutrophils. This hypothesis is based on previous evidence showing that: (a) C5a/C5aR1 signaling directly triggered neutrophil activation (e.g., granule enzyme release and superoxide anion production/respiratory burst) in several pathological conditions (66–71); (b) C5aR1 signaling induced neutrophils to degranulate — with increase in CD66 expression — in sepsis models (72); (c) C5a levels in the soluble fraction of sputum correlated positively with markers associated with worse cystic fibrosis lung disease, including NE levels, MPO activity, and DNA concentration (73). Additionally, our finding that C5a levels in the BAL of patients with COVID-19 correlated with degranulated/hyperactivated neutrophils also supports this hypothesis. Among the downstream mechanisms by which activated neutrophils might participate in the pathophysiology of COVID-19, the production of NETs is one of the most described (43, 44). For instance, we and others have previously shown that in the lungs of patients with COVID-19, SARS-CoV-2 directly triggers NET-dependent lung immunopathology (46, 74–76). We also found that hyperactivated neutrophils in the BAL from patients with COVID-19 are enriched for NET-related genes (34). Moreover, data from our lab also showed that Tg-infected mice treated with NETs-degrading DNase ameliorated lung pathology (46). Our present data showed that the inhibition of C5aR1 signaling in myeloid cells reduced the levels of NETs in the lung of SARS-CoV-2–infected mice. Corroborating this, we also found that C5a alone, via C5aR1, was able to induce lung inflammation/pathology in a NETs-dependent manner in vivo. These results raised the question of whether the C5a/C5aR1 signaling was driving NETs formation in a direct or indirect manner. In this context, we found that a low dose of C5a was able to promote NETs formation by naive human neutrophils in vitro, in a C5aR1 dependent manner. Mechanistically, C5a-triggered NETs in human neutrophils seem to be dependent on the PAD4/ROS canonical pathway. These findings are in agreement with evidence that plasma from patients with COVID-19 triggers NETs formation by human naive neutrophils, and this process was reduced by inhibition of C5aR1 signaling (77). Although these data strongly indicate that C5a/C5aR1 signaling directly causes NETs formation and this mechanism is important for the inflammatory activity of this signaling, we cannot exclude an indirect effect of C5a on NETs formation. In addition, we have shown that in vitro infection of naive human neutrophils with SARS-CoV-2 promoted NETs formation; this effect was dependent on the replication process, although the replication was not completed due to abortive replication (46). Herein, we also found that C5a enhances NETs formation by human neutrophils infected with SARS-CoV-2. These results suggest that in the lungs of patients with COVID-19 (and SARS-CoV-2–infected mice) the presence of infected neutrophils and higher levels of C5a might amplify the NETosis process. Although our data indicate the importance of C5aR1 signaling in neutrophils, which trigger NETs, that, in turn, contribute to the pathophysiology of COVID-19, it is noteworthy that in Tg^cKO^ mice, C5aR1 signaling is also interrupted in macrophages/monocytes (78). Therefore, we cannot exclude that part of the protective phenotype observed in the infected Tg^cKO^ mice would be due to inhibition of C5aR1 signaling in those cells, and that, indirectly, this might also affect NETs production.

Targeting C5aR1 signaling in SARS-CoV-2-infected mice, aside from inhibiting NETs formation in the lungs, also reduced the increase in the levels of proinflammatory cytokines/chemokines. This raised the question of whether NETs formation was intermediate to this process. Notably, our current data showing that lung inflammation promoted by C5a instillation, including increased chemokines levels, was prevented by NETs degradation favor this possibility. However, since we do not have the entire time course of C5a-induced lung inflammation, we could not exclude the possibility that C5a might induce an initial release of cytokines that in turn promote NETs. Subsequently, NETs could amplify the inflammatory process by promoting tissue damage and additional cytokine/chemokine production. In fact, there is evidence that NETs may amplify inflammation through tissue damage (78–81), including triggering direct cytokine/chemokine production (79, 80). Based on our data and previous data from the literature, our current hypothesis is that SARS-CoV-2 infection may trigger initial production of some cytokines and chemokines that promote neutrophil infiltration. At the local of infection in the lungs, neutrophils are activated by C5a to produce NETs, which may also synergize with virus infection and which promote tissue damage and could also amplify the inflammatory process.

Our data further reinforce the possibility of the effective use of inhibitors of C5a/C5aR1 signaling for the treatment of COVID-19. In fact, clinical results show that inhibition of C5a reduced COVID-19 hyper inflammation and improved lung function (25–27). Notably, a Phase 3 clinical study has shown that treatment of patients with severe COVID-19 with Vilobelimab, an anti-C5a monoclonal antibody, significantly reduced mortality (28). The hypothesis that the blockage of C5aR1 signaling would be beneficial to COVID-19 may open another important question related to secondary infections that are extremely common in patients with COVID-19 and are a critical threat in the current treatments targeting the immune response (81–85). Although inhibition of C5 by neutralizing antibodies has been associated with increased risk of bacterial infection due to the inhibition of the formation of the membrane attack complex, the selective targeting of C5a/C5aR1 signaling may avoid harmful anaphylatoxin-induced effects (86, 87). In fact, inhibition of C5aR1 signaling reduced the consequences of exacerbated bacterial infection such as is observed in sepsis (88–91). These studies gave support for the hypothesis that C5a/C5aR1 signaling is more important for immunopathology (tolerance) than for immune defense against infections (resistance).

Overall, our study provides direct evidence of the detrimental role of C5a/C5aR1 signaling for lung immunopathology in COVID-19 infections. It also provides the molecular mechanism by which C5aR1 signaling, especially in neutrophils via NETs-dependent lung pathology, mediates COVID-19 pathophysiology. In conclusion, our study confirms that inhibition of C5aR1 signaling, for example by orally active allosteric inhibitors, could be an alternative therapeutic against this disease.

## Methods

### COVID-19 mouse model.

K18-hACE2 transgenic (Tg) mice (B6.Cg-Tg(K18-ACE2)2Prlmn/J, catalog number 034860) and *Lyz2*^Cre/Cre^ (B6.129P2-Lyz2tm1(cre)Ifo/J, catalog number 004781) mice were purchased from the Jackson Laboratory. *C5ar1*^fl/fl^ mice, which also express eGFP under the C5aR1 promoter, were donated by Jörg Köhl (92) To generate Tg^cKO^ and Tg^fl/fl^, which were littermate controls, Tg mice were bred with *Lyz2*^Cre/0^*C5ar1*^fl/fl^ mice. Local colonies of transgenic mice were established and maintained at the Animal Care Facility of Ribeirão Preto Medical School, University of São Paulo. Food and water were available ad libitum and mice were kept in a controlled light-dark cycle. For COVID-19 induction, the animals received intranasal inoculation of SARS-CoV-2 (2 × 10^4^ PFU), which presents disease signs and lung pathology consistent with human disease. The manipulation of these animals was performed in a Biosafety Level 3 (BSL3) facility.

### Human and mouse C5a, factor Bb, and C3a level quantification.

The C5a, factor Bb, and C3a levels were determined in the BAL fluid and plasma from 16 critically ill adult patients with COVID-19, all having been in the intensive care unit (ICU) for under 20 days, and 16 patients with influenza, as a non–COVID-19 viral pneumonia cohort. Both patient cohorts have been described previously (29). C5a, factor Bb, and C3a ELISA assays were performed using, respectively, the human complement component C5a duoset ELISA kit from R&D Systems (DY2037), the MicroVue Bb plus fragment EIA kit from Quidel (A027), and the Complement C3a human ELISA kit from Thermo Fisher Scientific (BMS2089). All assays were performed according to the manufacturer’s instructions.

For mice, lung homogenate was obtained and the supernatant was collected. ELISA assays were performed to detect the concentration of C5a, factor Bb, and C3a using kits from R&D Systems (catalog numbers DY2150, NBP2-75243, and CTK-148, respectively), according to the manufacturer’s instructions.

### Virus stock production.

SARS-CoV-2 (Brazil/SPBR-02/2020 strain) was provided by Edison Luiz Durigon (ICB-USP, Sao Paulo, Brazil). The virus was propagated and titrated in Vero E6 cells in a BSL3 laboratory at the Center for Virus Research, Ribeirao Preto Medical School (Ribeirao Preto, Brazil). Cells were cultured in DMEM medium (Corning) supplemented with 10% FBS (GE Life Sciences) and antibiotic/antimycotic (Penicillin 10,000 U/ml; Streptomycin 10,000 μg/ml; Sigma-Aldrich). The viral inoculum was added to Vero cells in DMEM (FBS 2%) incubated at 37 °C with 5% CO_2_ for 48 hours. The cytopathogenic effect was observed under a microscope. A cell monolayer was collected and the supernatant was stored at –70ºC. Virus titration was performed by calculating the PFUs. The supernatant of noninfected cells was used for the control of the experiments (mock groups).

### Drugs and pharmacological treatment in vivo.

For in vivo experiments, we used DF2593A (3 mg/kg orally [p.o.]), a selective C5aR1 antagonist (41). For the COVID-19 mouse model, the drug was administered 1 hour before or 24 hours after SARS-CoV-2 inoculation and daily after infection. We assessed the daily clinical scores (Supplemental Table 1) and body weight of each animal. We also evaluated the oxygen saturation prior to the infection and daily after infection using a mouse pulse oximeter (MouseOx Plus, Starr Life Sciences). At 5 days after infection, lungs from mock and SARS-CoV-2–infected mice were collected. Lung lobules were collected, harvested, and homogenized in PBS with steel glass beads. The homogenate was added to TRIzol reagent (1:1; Invitrogen), for posterior viral titration via reverse transcription–quantitative PCR (RT-qPCR)**,** or to lysis buffer (1:1), for the ELISA assay, and stored at –70°C. In another cohort experiment, the left lung was collected in paraformaldehyde (PFA 4%; Millipore) for posterior histological assessment.

### In vitro SARS-CoV-2 infection.

Vero E6 cells (1 × 10^5^) were pretreated with DF2593A at 0.01; 0.1; 1.0; and 10.0 μM for 1 hour before SARS-CoV-2 infection at 37ºC. Cells were infected at a multiplicity of infection (MOI) of 1.0 with an infectious clone SARS-CoV-2 or the noninfected supernatant (mock) with infection media to evaluate viral load by RT-qPCR, 24 hours after infection. The treatment was performed in technical quadruplicate.

### SARS-CoV-2 viral load.

SARS-CoV-2 detection was performed with primer-probe sets for 2019-nCoV_N1 and N2 (Integrated DNA Technologies), according to the US CDC protocol by RT-qPCR, using total nucleic acids extracted with Trizol reagent from cell pellet or lung tissue to determine the genome viral load. All RT-qPCR assays were performed using the Viia 7 Real-time PCR System (Applied Biosystems). A standard curve was generated in order to obtain the exact number of copies in the tested sample. The standard curve was performed using an amplicon containing 944 bp cloned in a plasmid (PTZ57R/T CloneJetTM Cloning Kit, Thermo Fisher Scientific), starting in the nucleotide 14 of the gene N1. To quantify the number of copies, a serial dilution of the plasmid in the proportion of 1:10 was performed. Commercial primers and probes for the N1 gene and RNAse P (endogenous control) were used for quantification (2019-nCov CDC EUA Kit, Integrated DNA Technologies), following the CDC’s instructions.

### In vivo challenge with rmC5a.

C57BL6 8-week old male mice were treated with DNAse (Pulmozyme, 10 mg/kg, s.c.) twice before the challenge with rmC5a (400 nM) by intratracheal instillation (45), with treatment administered 24 hours and 1 hour before rmC5a. Eight hours after the challenge, lungs were collected and fixed in PFA 4% for subsequent histological analysis. Five-micrometer slices were submitted to H&E staining, and images were taken under a brightfield microscope. In another set of animals, we performed the same experiment and collected lungs for ELISA assay. The sandwich ELISA method was performed to detect the concentration of cytokines and chemokines using kits from R&D Systems (DuoSet), according to the manufacturer’s instructions. The following targets were evaluated: CCL2, CCL3, CCL4, CXCL1, and IL-6.

### Re-analysis of single-cell RNA-Seq data sets.

We reanalyzed single-cell transcriptomic data from BAL fluid cells from patients with severe COVID-19 and their respective control groups (31, 33). The data set was downloaded and the RDS file was imported into R environment version v4.04 and Seurat v4.1.1 by filtering genes expressed in at least 3 cells and more than 200 unique molecular identifiers (UMI) counts per cell. For the preprocessing step, outlier cells were filtered out based on 3 metrics: library size less than 60,000; number of expressed genes between 200 and 7,500; and mitochondrial percentage expression under 20. The top 3,000 variable genes were then identified using the vst method using the FindVariableFeatures function. Percent of mitochondrial genes was regressed out in the scaling step, and principal component analysis (PCA) was performed using the top 3,000 variable genes with 40 dimensions. Additionally, a clustering analysis was performed on the first 7 principal components using a resolution of 2 followed by t-distributed stochastic neighbor embedding (tSNE), a dimensionality reduction technique for data visualization. Then, differential gene expression analysis was performed using FindAllMarkers function with default parameters to obtain a list of significant gene markers for each cluster of cells. To account for the frequency of cells expressing C5AR1, we filtered cells with raw counts of C5AR1 of more than 0. The data set generated by authors is publicly available at the EGA, European Genome-Phenome Archive database (EGAS00001004717 accessible at https://ega-archive.org/studies/EGAS00001004717); or at https://covid19-balf.cells.ucsc.edu/

### Human lung samples from autopsies.

We performed adapted minimally invasive autopsies from 4 fatal COVID-19 cases (93). (Supplemental Table 2). Briefly, a mini-thoracotomy (3 cm) was done under the main area of lung injury identified by earlier ultrasound. The lung parenchyma was clamped by Collins Forceps, cut, and fixed in 10% buffered formalin (Sigma-Aldrich).

### H&E staining and lung pathology.

Lung slices of 5 μm were fixed with PFA 4%, paraffin-embedded, and submitted for H&E staining. The morphological analysis was based on the Standards for Quantitative Assessment of Lung Structure published by the American Thoracic Society/European Respiratory Society (ATS/ERS). Briefly, a systematic uniform random sampling of the lungs was performed. Considering uniform lung inflation and fixation in 10% buffered formalin, 10 high-power field photographs were taken of the H&E slides of each case followed by selection of the septal component and determination of its area versus total area using the Image Pro Plus software. The ratio between total septal area and the total lung area was expressed as area fraction (%). The mean area fraction values between the 10 high-power field photographs from each animal were used for statistical comparison between groups and for graphical representation. Additional histological evaluation was performed by an expert pathologist.

### Immunostaining and confocal microscopy.

Lung samples from COVID-19 autopsies or Tg^fl/fl^-infected mice were fixed with PFA 4%. After dehydration and paraffin embedding, 5-μm sections were prepared. The slides were deparaffinized and rehydrated by immersing the through Xylene (Labsynth) and 100% Ethanol (Labsynth) for 15 minutes with each solution. Antigen retrieval was performed with 1.0 mM EDTA (Labsynth) 10 mM Trizma-base (Sigma-Aldrich), pH 9.0 at 95°C for 30 minutes. Afterward, endogenous peroxidase activity was quenched by incubation of the slides in 5% H_2_O_2_ in methanol (Millipore) at RT for 20 minutes. After blocking with IHC Select Blocking Reagent (Millipore) at RT for 2 hours, primary antibodies were incubated overnight at 4°C. Antibodies included mouse monoclonal anti-C5aR1 (clone: S5/1; Millipore, MABF1980; 1:50), rabbit polyclonal anti-IBA1 (FUJIFILM Wako Pure Chemical Corporation, 016-20001; 1:200), rabbit polyclonal anti-NE (Abcam, ab68672; 1:100), goat polyclonal anti-MPO (R&D Systems, AF3667, 1:100) and rabbit polyclonal, anti-histone H3 (H3Cit; Abcam, ab5103; 1:100). The slides were washed with TBS-T (Tris-Buffered Saline with Tween 20) and incubated with secondary antibodies alpaca anti-mouse IgG Alexa Fluor 594 (Jackson ImmunoResearch, 615-585-214; 1:1,000), donkey anti-goat IgG Alexa Fluor 488 (Abcam, ab150129), alpaca anti-rabbit IgG Alexa Fluor 488 (Jackson ImmunoReseach, 611-545-215; 1:1,000) and alpaca anti-rabbit IgG AlexaFluor 594 (Jackson ImmunoReseach, 611-585-215; 1:1,000). Autofluorescence was quenched using the TrueVIEW Autofluorescence Quenching Kit (Vector Laboratories, SP-8400-15). The percentage of cells expressing C5aR1 was determined by colocalization between Iba1 (macrophage) or NE (neutrophil) with C5aR1 expression. Four randomized fields from 4 fatal COVID-19 cases or Tg^fl/fl^-infected mice were analyzed.

For NETs detection in vitro, neutrophils were plated in 24-well plates containing glass coverslips covered with 0.01% poly L-lysine solution (Sigma-Aldrich), fixed with PFA 4% at room temperature for 10 minutes, 2% BSA (Sigma-Aldrich) and 22.52 mg/ml glycine (Sigma-Aldrich) in Phosphate Buffer Saline + 0.1% Tween 20 (PBST) at room temperature for 2 hours. The coverslips were stained with rabbit polyclonal anti-Neutrophil Elastase (anti-NE; Abcam, ab68672; 1:500) and mouse monoclonal anti-MPO (2c7; Abcam, ab25989, 1:800). After this, samples were washed in PBS and incubated with secondary antibodies, including alpaca anti-mouse IgG AlexaFluor 488 (Jackson ImmunoResearch, 615-545-214; 1:1,000) and alpaca anti-rabbit IgG AlexaFluor 594 (Jackson ImmunoResearch, 611-585-215; 1:1,000). Slides were then mounted using ProLong diamond antifade mountant with DAPI (Thermo Fischer Scientific). Images were acquired by Axio Observer combined with LSM 780 confocal microscope (Carl Zeiss) at 630× magnification at the same setup of zoomed, laser rate, and scanned with 4 fields/image (tile scan function). NETs were quantified by the ratio between the total number of cells per field versus the number of cells under NETosis (cells with loss of nucleus segmentation or cells in the process of releasing chromatin in networks) (NETosis = (Cells / Cells in NETosis) × 100). Images were acquired and analyzed using Fiji by Image J.

### Apoptosis TUNEL assay.

Frozen lung tissue slices were used for the detection of apoptotic cells in situ with Click-iT Plus TUNEL Assay Alexa Fluor 488, according to the manufacturer’s instructions (Thermo Fisher Scientific; cat. C10617). The slides were counterstained with Vectashield Antifade Mounting Medium with DAPI. Images were acquired by microscope Leica DMI6000B (Leica microsystems) at 200× magnification at the same laser rate. Ten fields (322.8 μm, 2 each) of the left lung were analyzed using Fiji by Image J, which represents 75% of the total area of the left lung. The apoptosis quantification was performed as the percentage of TUNEL-positive cells from DAPI staining.

### Production of NETs by isolated human neutrophils.

Blood samples were collected from healthy controls by venipuncture for in vitro experiments. Neutrophils were isolated and purified by Percoll density gradient (72%, 63%, 54%, and 45%) (GE Healthcare). Isolated neutrophils were resuspended in RPMI 1640 medium (Corning). Neutrophil purity was over 95%, as determined by Rosenfeld’s Color Cytospin (Laborclin). A total of 1 × 10^6^ isolated neutrophils were attached to coverslips coated with poly L-lysine solution 0.1% (Sigma-Aldrich) incubated for 4 hours at 37°C for NET immunostaining.

In the first protocol, isolated human neutrophils were incubated with the PAD4 inhibitor CL-amidine (200 μM), with the ROS production inhibitor DPI (10 μM/mL), or with the C5aR1 antagonist DF2593A (1 μM) for 1 hour and, subsequently, challenged with rhC5a (3 nM; R&D Systems). The concentration of rhC5a was based on a concentration-response curve. Four hours after the challenge, supernatant was collected and stored at –80°C. Cells were collected on a coverslip and submitted to immunofluorescence for the visualization of NETs.

In the second protocol, neutrophils were incubated with mock supernatant, rhC5a (3 nM), or infected with SARS-CoV-2 (MOI = 1.0). One group of cells was incubated with SARS-CoV-2 and treated with rhC5a (3 nM). Four hours after the challenge, supernatant was collected and stored at –80°C. Cells were collected on a coverslip and submitted to immunofluorescence for the visualization of NETs.

### NETs quantification in the lung tissue.

The 96-well black plates were coated with anti-MPO antibody (Thermo Fisher Scientific, PA5-16672; 1:1000) overnight at 4ºC. Subsequently, the plate was washed with PBS + 0.1% Tween 20 and blocked with 2% BSA for 2 hours at room temperature. The lung tissue homogenates were obtained and centrifuged at 10,000*g* at 4°C for 10 minutes. The supernatant was collected and incubated overnight at 4°C. On the third day, MPO-bound DNA (NETs) was quantified using the Quant-iT PicoGreen kit (Invitrogen) as previously described (45).

### Flow cytometry analysis.

Lung tissue was harvested and digested with type 2 collagenase (1 mg/ml, Worthington) for 45 minutes at 37ºC to acquire cell suspensions. Total lung cells (1 × 10^6^) were then stained with Fixable Viability Dye eFluor 780 (Invitrogen, 65–0865-14; 1:1000) and monoclonal fluorochrome-stained antibodies specific for CD45 (BD Pharmingen; clone 30F-11, 553080; 1:200), CD11b (Biolegend; clone M1/70, 101212; 1:200), Ly6G (Biolegend; clone 1A8, 127606; 1:200), CCR2 (Biolegend; clone SA203G11, 150605; 1:200), and Ly6C (eBioscience; clone HK1.4, 45-5932-82; 1:200) for 30 minutes at 4°C. Data was acquired on FACSVerse flow cytometer (BD Biosciences) and analysis was performed using FlowJo (TreeStar) software. Gating strategies for flow cytometry analysis are schematically represented in (Supplemental Figure 11).

### Cytokine and chemokine quantification.

Lung homogenate was added to the RIPA buffer in the proportion of 1:1, and then centrifuged at 10,000*g* at 4°C for 10 minutes. The supernatant was collected and stored at –70°C until use. The sandwich ELISA method was performed to detect the concentration of cytokines and chemokines using kits from R&D Systems (DuoSet), according to the manufacturer’s instructions. The following targets were evaluated: CCL2, CCL3, CCL4, CXCL1, CXCL2, IFN-β, IL-6, IL-10, and TNF.

### Statistics.

Statistical significance was determined by either 1 or 2-tailed unpaired and paired Student *t* tests, 1-way or 2-way ANOVA followed by Bonferroni’s posthoc test. Spearman correlation analysis was performed by calculating a repeated measures correlation coefficient (*r* value) and was plotted utilizing a simple linear regression line. *P* < 0.05 was considered statistically significant. Statistical analyses and graph plots were performed and built with GraphPad Prism 9.3.1 software.

### Study approval.

Experimental procedures were performed in accordance with the guide for the use of laboratory animals of the University of Sao Paulo and approved by the ethics committee under protocol numbers 021/2021.

The use of human samples was approved by the Ethics Committee of the University Hospitals Leuven under the protocol S63881. Written informed consent was obtained from all study participants or their legal representatives according to the ethical guidelines of the Declaration of Helsinki. Minimally invasive autopsies were approved by the Ribeirão Preto Medical School Ethical Committee (protocol no. 4.089.567).

### Data availability.

The data supporting the findings are available within the paper and its supplementary information files or otherwise stated.

## Author contributions

TMC, BMS, FPV, and GFG designed, performed experimental work, analyzed data, and prepared the manuscript. BMS, FPV, GFG, DBC, DCN, GCMC, and GVLS performed experimental work related to FACS and analyzed data. BMS, FPV, and GFG performed experiments related to infection and harvested tissue. SC and FB performed experiments with BAL samples, including ELISA assays. BMS and GFG performed ELISA assay in mouse samples. GVLS, IMSC, PVM, HIN, and DL performed the single-cell transcriptome analysis. AHS, JCS, NLB, and CMS performed neutrophil isolation and NETs quantification. FPV and JCS performed immunostaining and confocal analysis. FPV performed TUNEL assay. SSB and ATF contributed to lung autopsy analysis and histopathological analyses. BMS, SD, IMP, DMJ, and RM performed SARS-CoV-2 viral load and viral stock. BMS performed in vitro infections. AUQ and JK performed Tg^cKO^ mice generation. PLJ, RDO, PP, EW, LV, SF, and JW contributed to the collection of clinical specimens and demographic and clinical characteristics analysis from COVID-19 and influenzas patients. TR, AS, DSZ, LOL, JCAF, EA, LDC, LB, AA, and FQC performed experiments and provided important scientific comments. MA, LB, EA, and FQC provided critical materials and comments. TMC designed, directed, and supervised the study, interpreted data, and wrote the manuscript. All authors reviewed the manuscript and provided final approval for submission. The order of cofirst authors was based on the order that each individual joined the project.

## Supplementary Material

Supplemental data

## Version 1 04/27/2023

In-Press Preview

## Version 2 06/15/2023

Electronic publication
