# Supplementary material for: C5aR1 signaling triggers lung immunopathology in COVID-19 through neutrophil extracellular traps
Source: J Clin Invest. 2023 Jun 15;133(12):e163105. doi: 10.1172/JCI163105 (PMC10266787; doi:10.1172/JCI163105)
Supplement: Supplemental data [file jci-133-163105-s255.pdf]

Supplementary Materials for

C5aR1 signaling triggers lung immunopathology in COVID-19 through neutrophil extracellular traps

Bruna M. Silva[1,2#], Giovanni F. Gomes[1#], Flavio P. Veras[1#], Seppe Cambier[3], Gabriel V. L. Silva[1,2], Andreza U. Quadros[1], Diego B. Caetité[1,2], Daniele C. Nascimento[1,2], Camilla M. Silva[1,2], Juliana C. Silva[1,2,], Samara Damasceno[1,2], Ayda H. Schneider [1,2], Fabio Beretta[3], Sabrina S. Batah[4], Icaro M. S. Castro[5], Isadora M. Paiva[1,2], Tamara Rodrigues[6], Ana Salina[1,6], Ronaldo Martins[3,4], Guilherme C.M. Cebinelli[1,2], Naira L. Bibo[1,2], Daniel M. Jorge[3,4], Helder I. Nakaya[5], Dario S. Zamboni[1,6], Luiz O. Leiria[1,2], Alexandre T. Fabro[4], José C. Alves-Filho[1,2], Eurico Arruda[6,7], Paulo Louzada-Junior[1,8], Renê D. Oliveira[1,8], Larissa D. Cunha[1,6], Pierre Van Mol[9], Lore Vanderbeke[10], Simon Feys[11], Els Wauters[12], Laura Brandolini[13], Fernando Q. Cunha[1,2], Jörg Köhl[14,15], Marcello Allegretti[13], Diether Lambrechts[9], Joost Wauters[11,16], Paul Proost[3], Thiago M. Cunha[1,2]*

*Correspondence to: thicunha@fmrp.usp.br

This PDF file includes:

Supplementary Figures 1 to 11

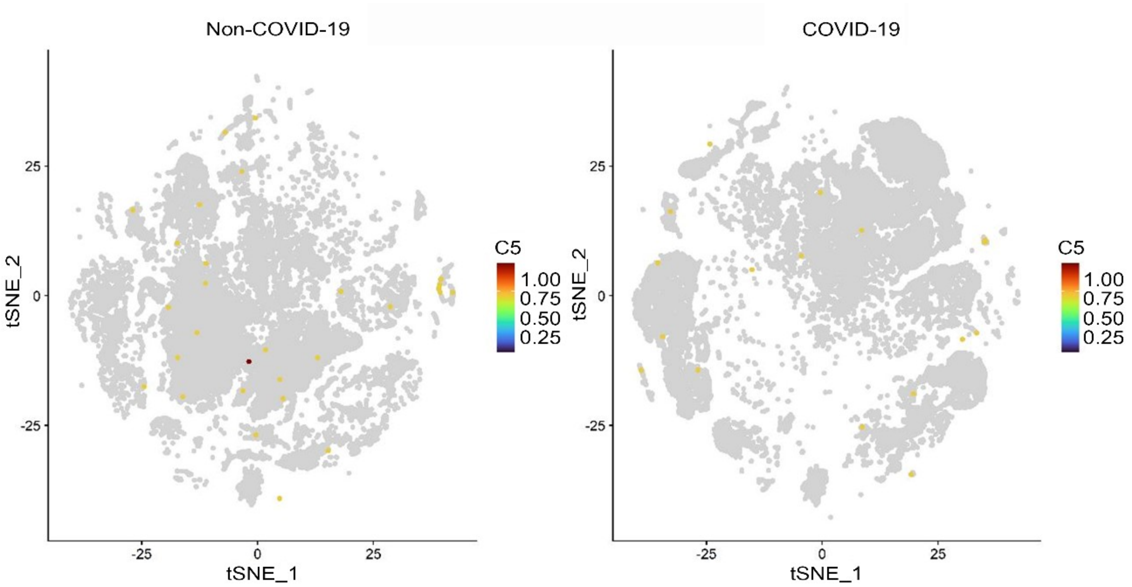

Supplementary Figure 1 - *C5* expression in the BAL fluid cells of COVID-19 patients. A previously published dataset was re-analyzed and the t-Distributed Stochastic Neighbor Embedding (t-SNE) analysis of total cells (65,166) from BAL fluid of non-COVID-19 pneumonia (n=13) and COVID-19 patients (n=22) is shown. Dot plots display the highlighted distribution of *C5* for each indicated cell population. The identification of cell populations in the tSNE map can be found in Figure 1F.

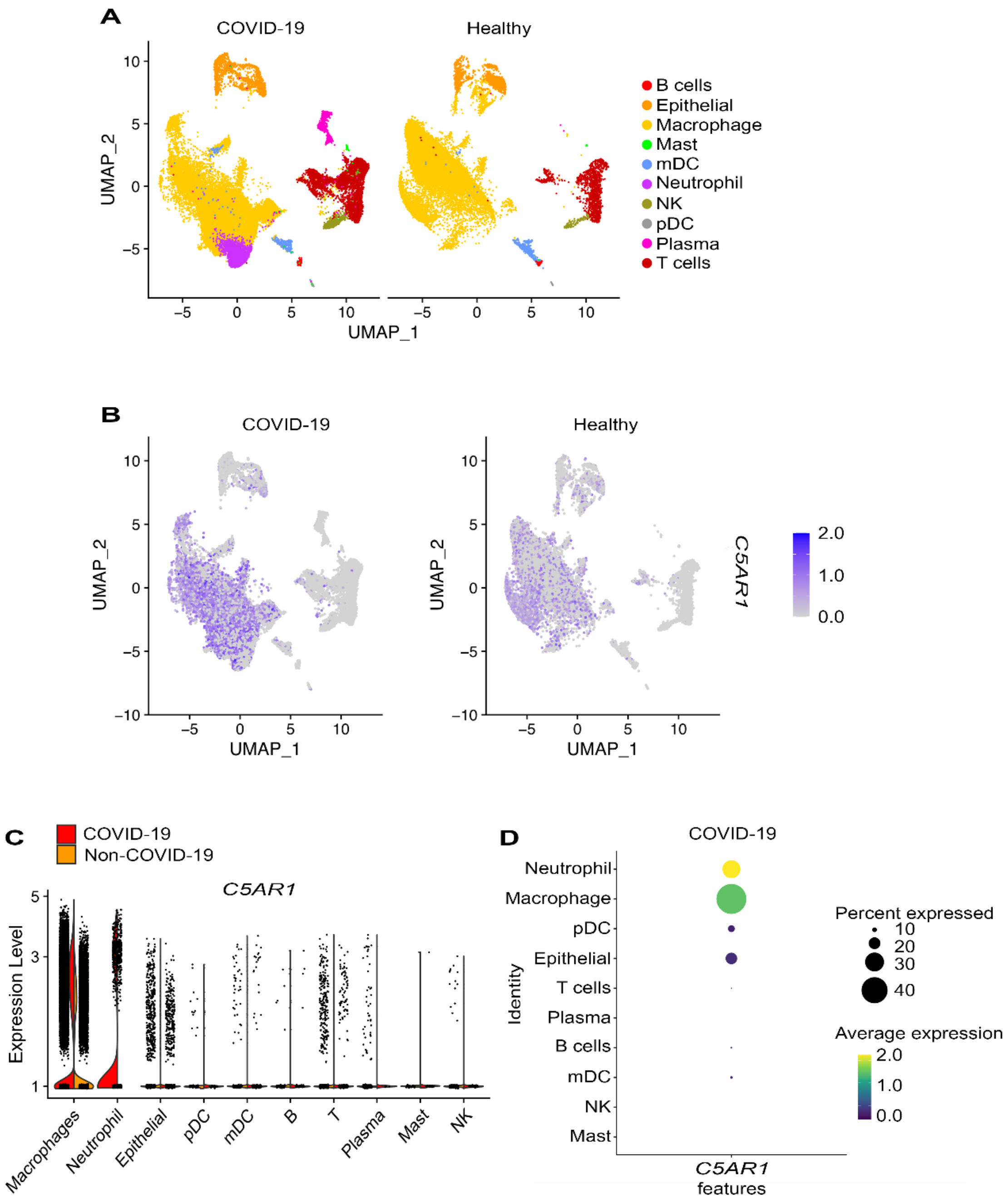

Supplementary Figure 2 - *C5AR1* expression in the BAL fluid cells of COVID-19 patients. (**A**) t-SNE analysis of total cells from BAL fluid from healthy controls (n=3) and severe COVID-19 patients (n=6). (**B**) Dot plots display the highlighted distribution of *C5AR1* for each indicated cell population. (**C**) Violin plots showing the expression levels of *C5AR1* in each type of cell. (**D)** The dot plot depicts the scaled and centered expression of an average cell in each cluster and therefore contains negative and positive values. The average expression reflects the mean expression of *C5AR1* in each cluster compared with all other cells.

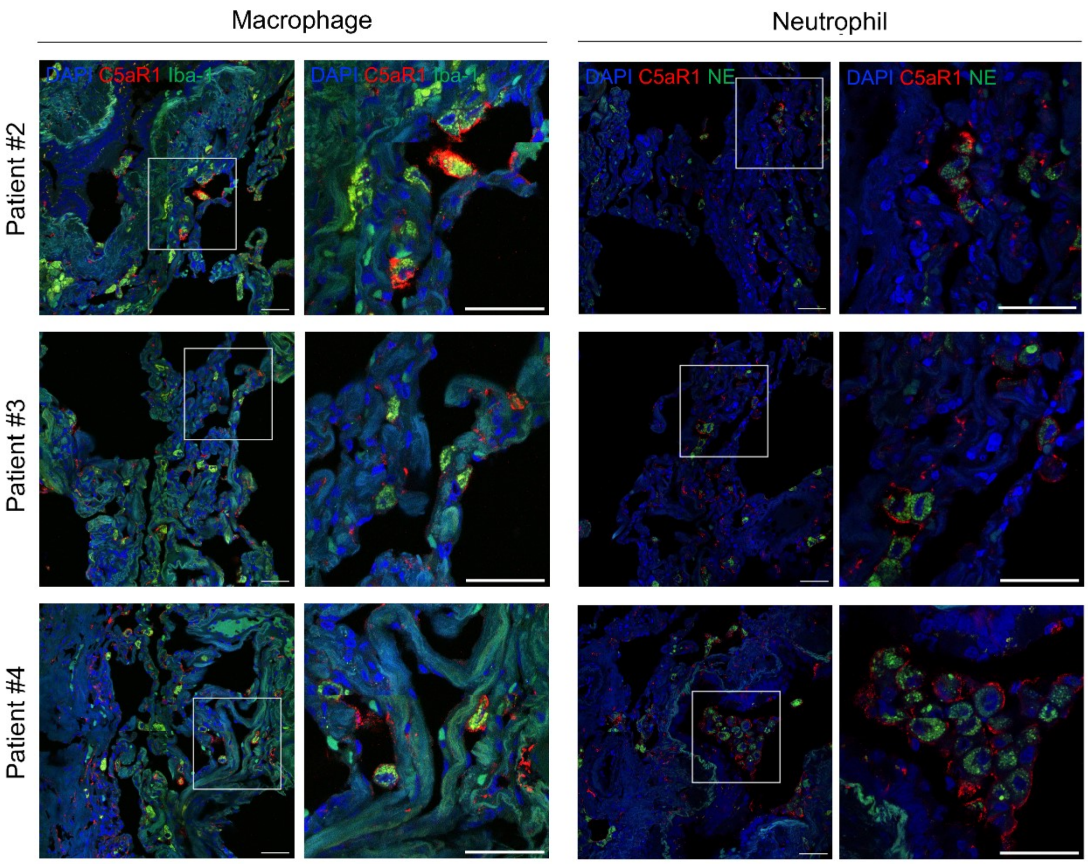

Supplementary Figure 3 - C5aR1 immunolabeling in neutrophils and macrophages in lung slices from patients with COVID-19. Representative confocal images of the presence of C5aR1 in macrophages (Iba-1) and neutrophils (NE) in the lung tissue from autopsies of 3 additional COVID-19 patients. Cells were stained for nuclei (DAPI, blue), Iba-1 or NE (green), and C5aR1 (red). Scale bar indicates 50 µm.

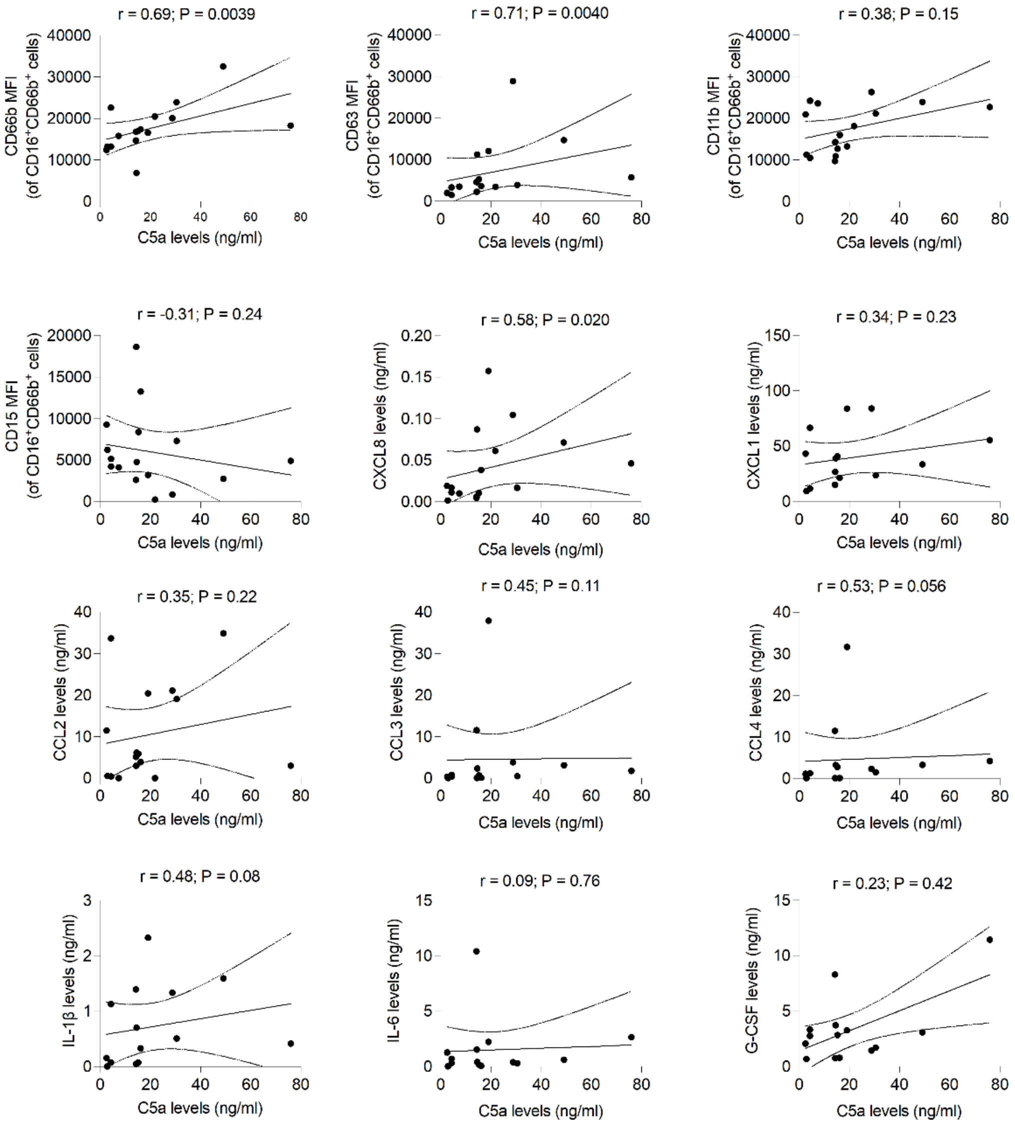

Supplementary Figure 4 – Correlation analyses between C5a and neutrophil markers/ inflammatory mediators in the BAL fluid from COVID-19 patients. Correlation analyses were performed with obtained levels of C5a (Fig. 1A) and the levels of different neutrophil membrane markers and inflammatory mediators that we found increased in the BAL fluid from COVID-19 patients determined in our previous study (29). For the neutrophil membrane markers CD66b, CD63, CD11b and CD15, the median fluorescence intensity (MFI) of neutrophils ($CD16^{+}CD66b^{+}$ cells) in the BAL fluid is shown. Data depicts Spearman's correlation coefficient (r) and P-value is depicted in each graph.

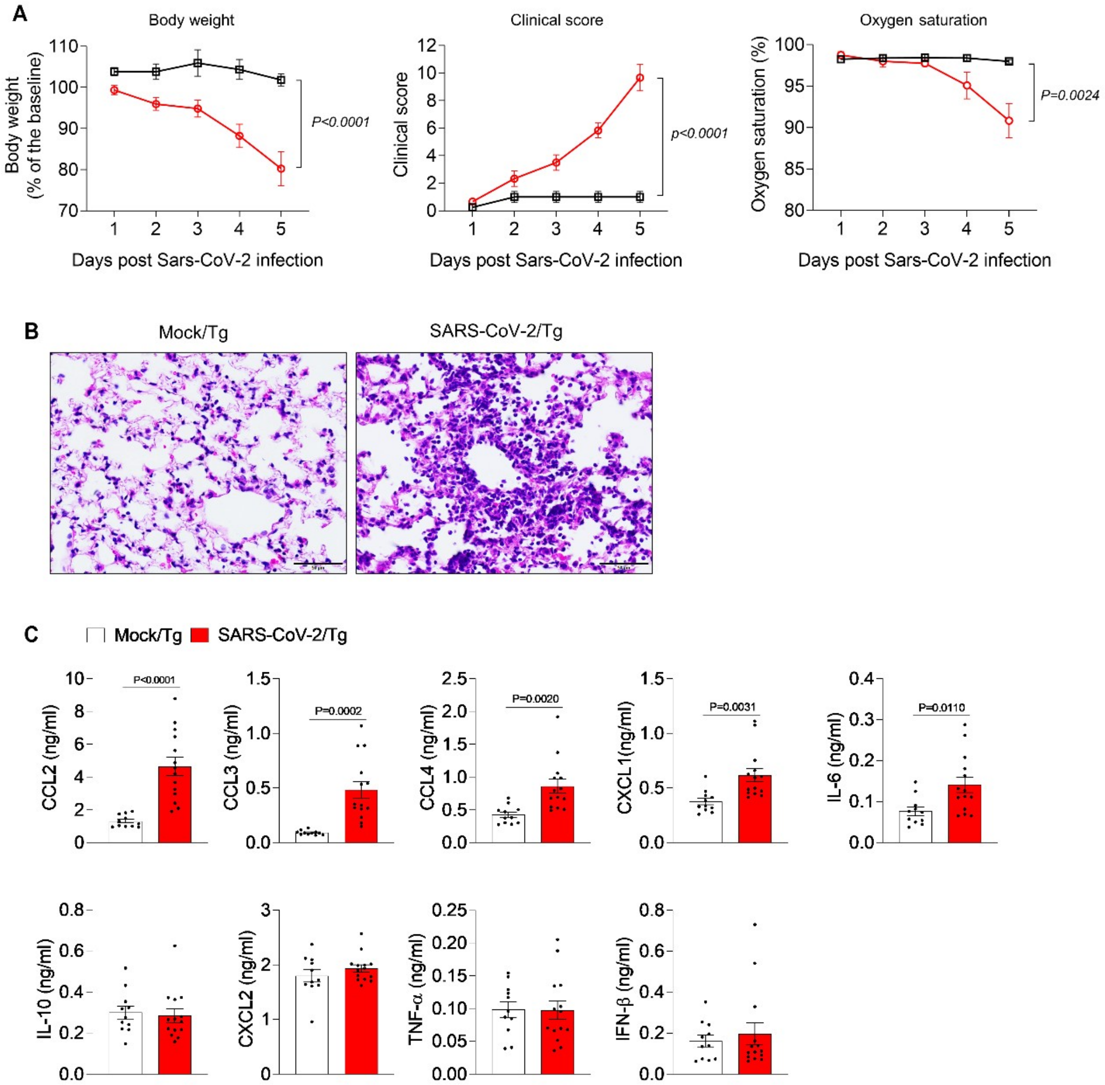

Supplementary Figure 5 - Immunopathology of COVID-19 mouse model. Tg mice were infected with SARS-CoV-2 (2 x 10^4 PFU, i.n.) on day 0. (**A**) Body weight and clinical score were measured daily post-infection (n=4-6). (**B**) Representative image of the lung tissue of the COVID-19 mouse model harvested at 5 dpi and stained with H&E. Mock-infected group was used as control. (**C**) ELISA assays were performed to detect CCL2, CCL3, CCL4, CXCL1, IL-6, IL-10, CXCL2, TNF-α and IFN-β levels in lung tissue of SARS-CoV-2 infected mice (n=14; pooled from two independent experiments). Mock was performed as control (n=11; pooled of two independent experiments). Data are shown as the mean ± S.E.M. P values were determined by two-way ANOVA followed by Tukey's multiple comparisons test (**A**) and unpaired Student *t*-test (**C**).

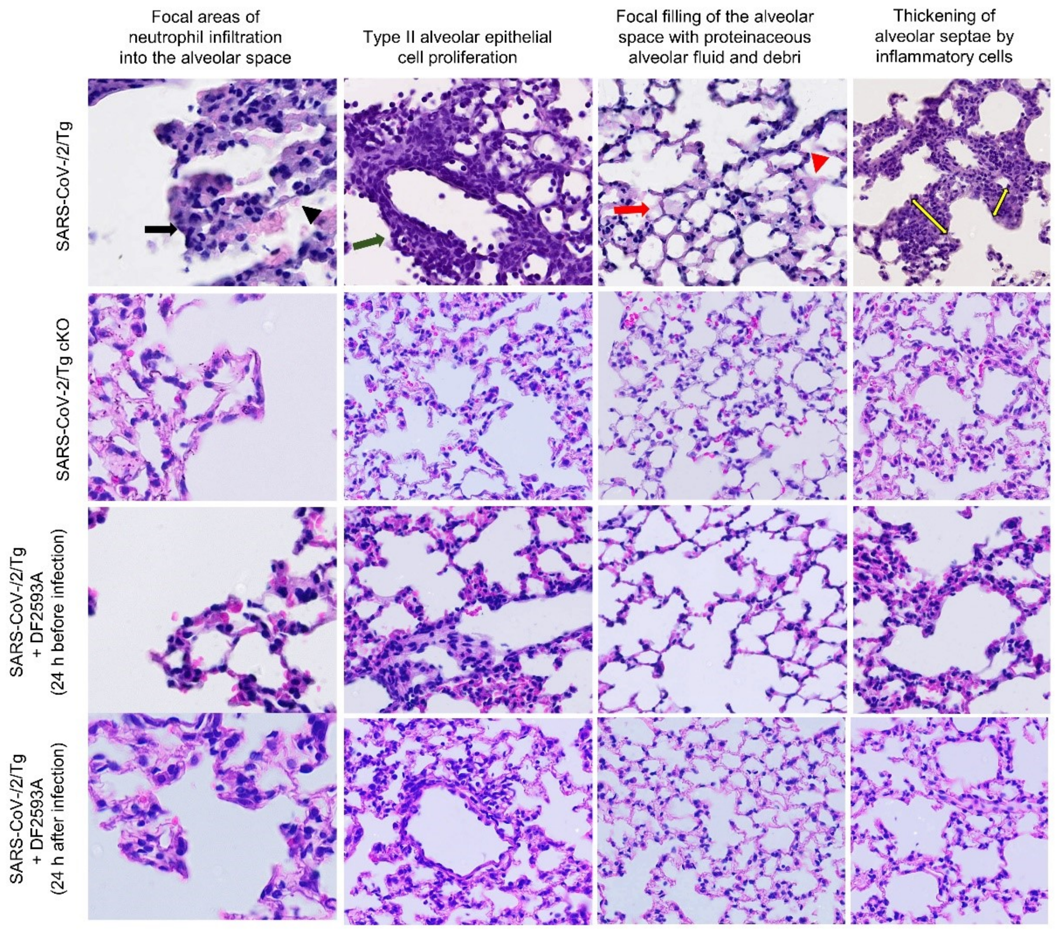

Supplementary Figure 6 - Pathological alterations in the lungs of the COVID-19 mouse model. Histological evidence of focal area of neutrophil infiltration into the alveolar space (black arrow), presence of blood cells in airspace (black arrowhead), type II alveolar epithelial cell proliferation (green arrow), focal filling of the alveolar space with proteinaceous alveolar fluid and debris (red arrow), focal type II alveolar epithelial cell injury (red arrowhead), and thickening of alveolar septae by inflammatory cells (yellow double-headed arrow in Tg mice infected with SARS-CoV-2. cTg-KO and DF2593A-treated mice did not present the same histological changes.

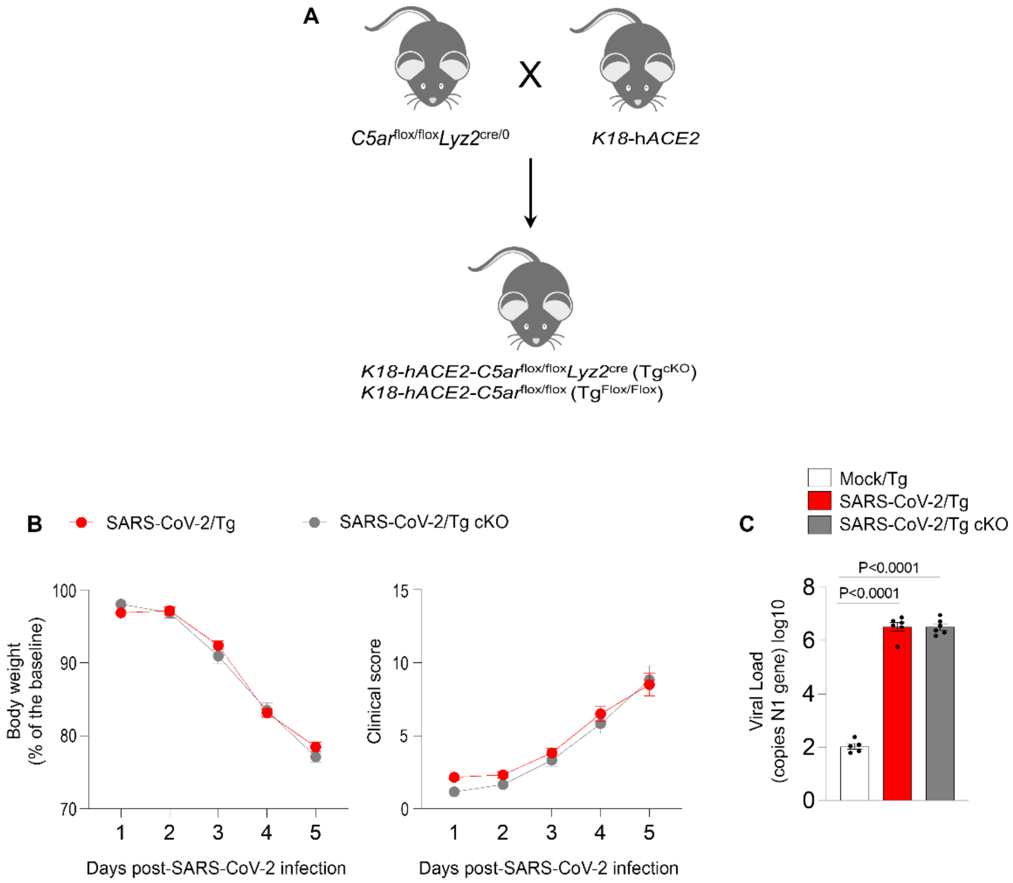

Supplementary Figure 7 - SARS-CoV-2-infected C5aR1-deficient K18-hACE2 mice. **(A)** Schematic representation of the generation of Tg^{cKO} mice and $Tg^{Flox/Flox}$ littermates. (**B**) Body weight and clinical score were measured daily (n=6/group) in $Tg^{Flox/Flox}$ and Tg^{cKO} mice infected with SARS-CoV-2 (2 x 10^4 PFU, i.n.). (**C**) Viral load in the lung tissue from infected $Tg^{Flox/Flox}$ and Tg^{cKO} mice (n=6/group) was evaluated by RT-PCR. Mock-infected group was used as control (n=5). Data are shown as mean ± S.E.M. P-values were determined by one-way ANOVA followed by Bonferroni's post hoc test (**C**).

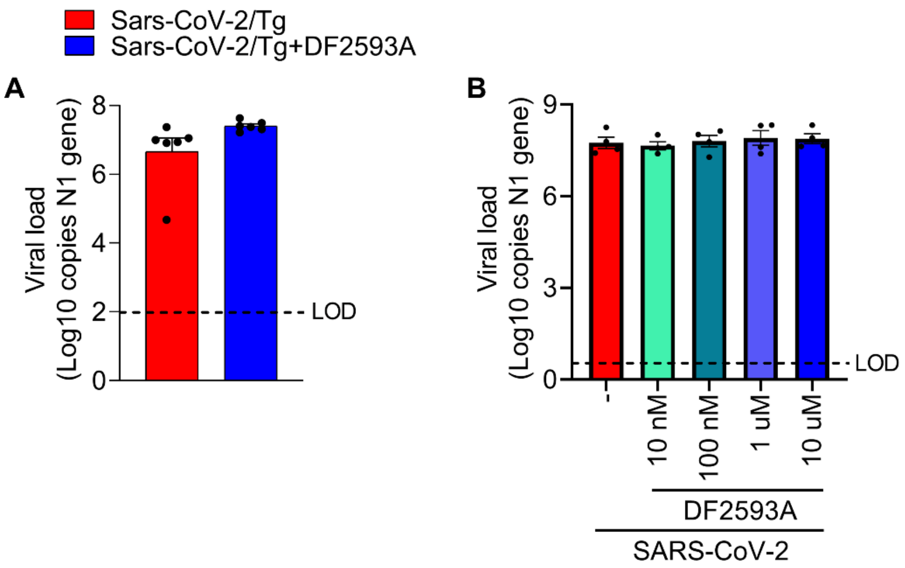

Supplementary Figure 8 – Effect of DF2593A treatment on viral load in the COVID-19 mouse model. (A) SARS-CoV-2 viral load in the lung tissue from Tg-infected mice treated with DF2593A (n=6) or vehicle (n=6). Mock-infected group was performed as control (n=6). (**B**) Vero E6 cells were pretreated with DF2593A (0.01-10 µM) followed by SARS-CoV-2 infection (MOI 1.0). Cells were lysed and the levels of SARS-CoV-2 were determined 24 h post-infection. The copy numbers of the N gene of SARS-CoV-2 were used to access the viral load by RT-PCR. Data are shown as the mean ± S.E.M. P-values were determined by one-way ANOVA followed by Bonferroni's post hoc test (**A** and **B**).

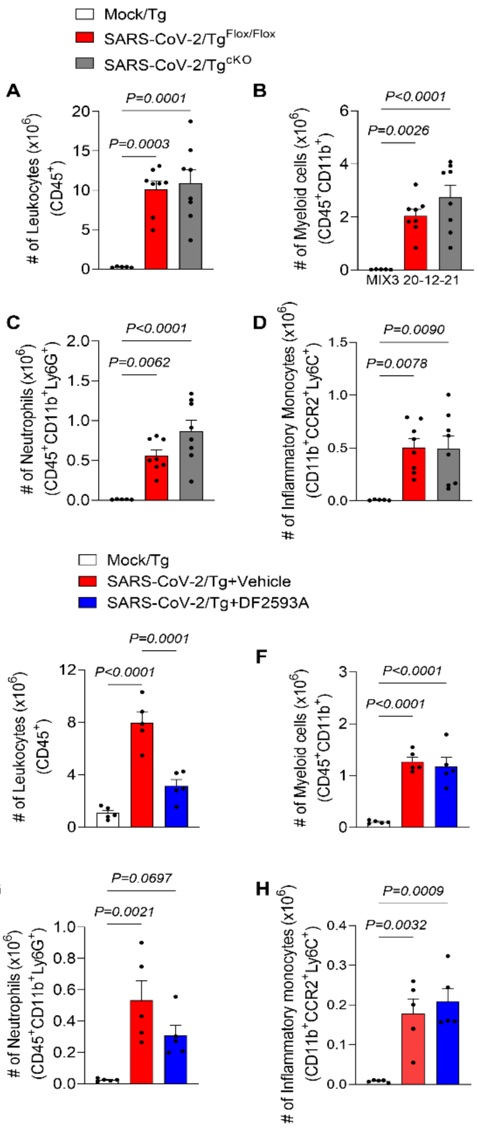

Supplementary Figure 9 - Leukocyte infiltration in the lung tissue of the COVID-19 mouse model. (**A**) Number of total leukocytes (CD45+ cells), (**B**) myeloid cells (CD45+CD11b+ cells), **(C)** granulocytes (CD45+CD11b+ Ly6G+ cells) and (**D**) inflammatory monocytes (CD45+CD11b+ CCR2+Ly6C+ cells) were determined by flow cytometry in the lung tissue from Tg^{cKO} or $Tg^{Flox/Flox}$ infected mice harvested at 5 dpi (n= 8/per group). Mock-infected group was used as control (n=5). Data are shown as the mean ± S.E.M. P-values were determined by one-way ANOVA followed by Bonferroni's post hoc test. (**E**) Number of total leukocytes (CD45+ cells), (**F**) myeloid cells (CD45+CD11b+ cells), **(G)** granulocytes (CD45+CD11b+Ly6G+ cells) and **(H)** inflammatory monocytes (CD45+CD11b+CCR2+Ly6C+ cells) were determined by flow cytometry in the lung tissue from Tg-infected mice treated with vehicle or DF2593A (n= 5/per group) harvested at 5 dpi. Mock-infected group was used as control (n=5).

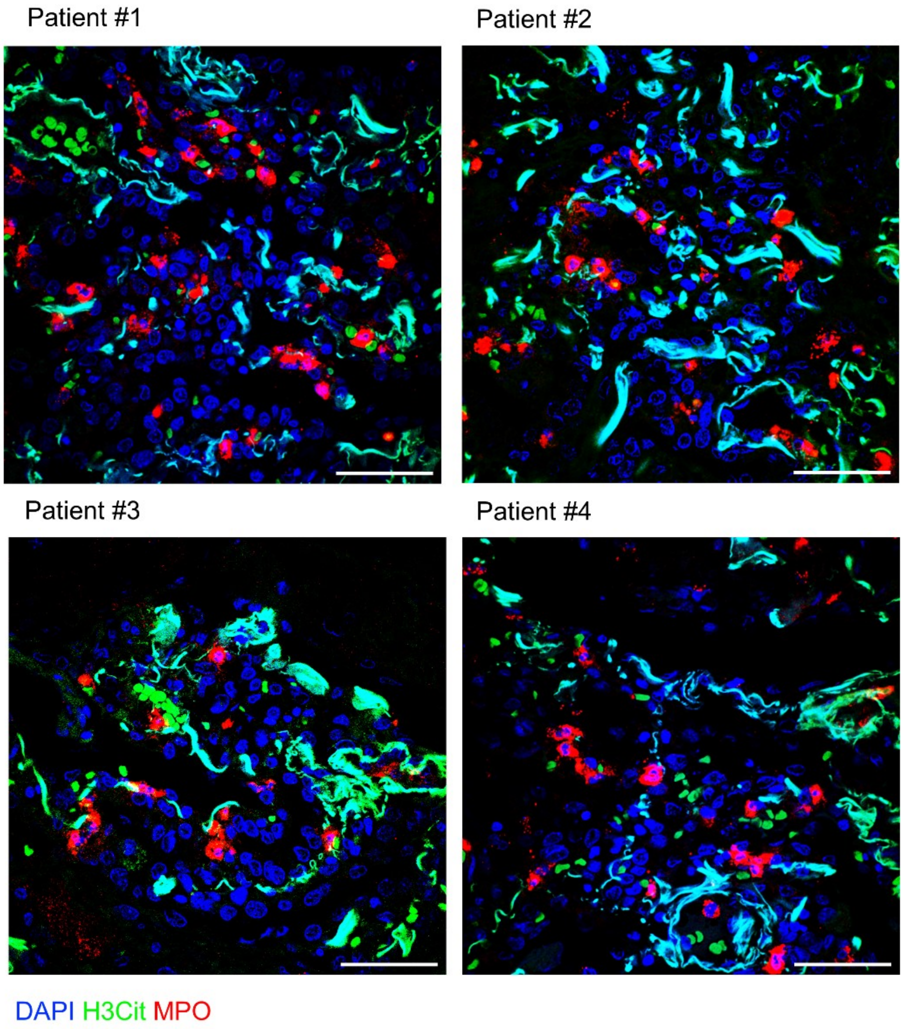

Supplementary Figure 10 - NETs production is increased in patients with COVID-19. Lung slices from four COVID-19 fatal cases. Representative images show nuclei (DAPI, blue), H3Cit (green), and MPO (red). Scale bar indicates 50 µm.

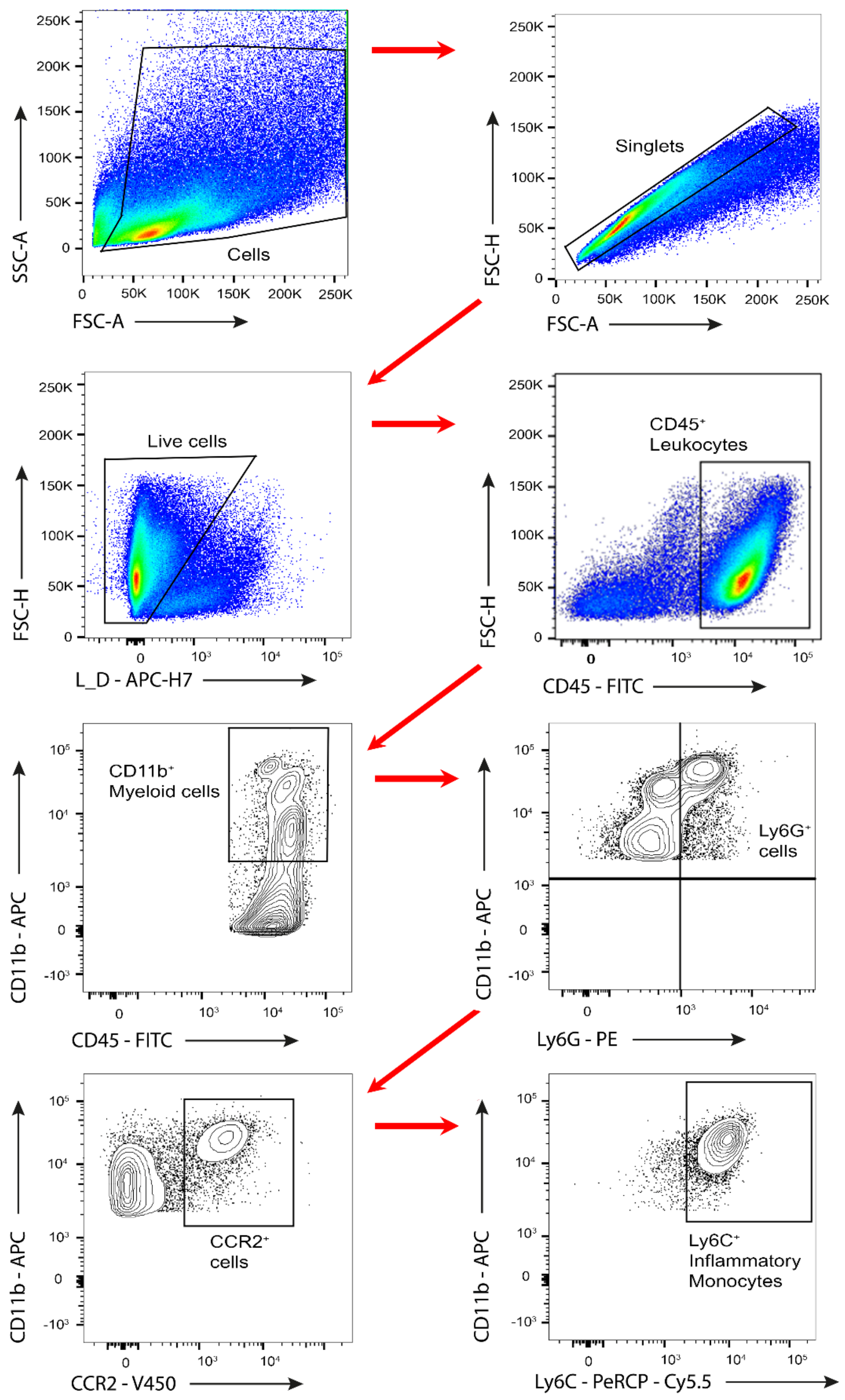

Supplementary Figure 11 – Representative gating strategies for flow cytometry analysis in the lung tissue. Gating strategies for different cell populations for all flow cytometry analyses.: **1)** Side scatter area (SSC-A) and forward scatter area (FSC-A); **2)** Doublet cells were excluded by forward scatter height (FSC-H) and FSC-A gating; **3)** Viable cells were identified using fixable viability dye and FSC-H gating; **4)** the number

of total leukocytes were identified as cells stained for $CD45^+$ among live cells. **5)** Myeloid cells were characterized as stained for $CD11b^+$ among $CD45^+$ live cells. **6)** Neutrophils were identified by $Ly6G^+$ inside $CD11b^+$ myeloid cells. **7)** Inflammatory monocytes were characterized by $Ly6C^+CCR2^+$ among $CD11b^+$ myeloid cells.

Supplementary Tables 1 and 2

Supplementary table 1

Table S1. Criteria of clinical sickness scores[1]

Clinical parameters	Degree	Score points
	Normal	0
	Mil<5%)	1
**Body weight loss**	6-10%	2
	11-15%	3
	16-20%	4
	>20%	5
**Appearance**	No piloerection	0
	Piloerection	1
	Alert	0
**Spontaneous behavior**	Slow moving	1
	Lethargic	2
	Immobile	3
	Normal	0
**Eyes**	Squinted	1
	Closed	2
	Quickly moves away	0
**Provoked behavior**	Slow to move away	1
	Do not respond	2
**Breathing**	Normal	0
	Elevated	1

[1] Adapted from Moreau et al. (Am. J. Trop. Med. Hyg., 2020) and Kumari et al. (Viruses, 2021).

Supplementary table 2

Table S2. COVID-19 patient characteristics

Demographics		%
Number	4	
Age (years)	69.75±13.84	
Hospital day	14.0±8.042	
Female	1	25%
**Comorbities**		
Hypertension	4	100%
Diabetes	3	75%
Obesity	1	25%
Lung disease	0	0%
History of smoking	2	50%
Heart disease	2	50%
Kidney disease	1	25%
Cancer	0	0%
Autoimmune diseases	0	0%
Immune deficiency	0	0%
**Laboratory findings**		
CRP (mg/dL)*	9.968±4.828	
D-Dimers (µg/mL)**	5.468±1.740	
LDH (U/L)#	1,053.0±878.9	
Ferritin (ng/mL)&	2,644±2,057	
Haemoglobin (g/dL)	12.58±2.457	
Neutrophils (cell/mm^3)	7,475±4,518	
Lymphocytes (cell/mm^3)	1,850±1,396	
Platelets (count/mm^3)	160,000±71,643	
**Medications**		
Antibiotics	4	100%
Heparin	4	100%
Antimalarial	0	0%
Oseltamivir	1	25%

Glucocorticoids	2	50%
**Respiratory status**		
Mechanical ventilation	4	100%
Nasal-cannula oxygen	4	100%
Room air	0	
pO_2	59.98±17.50	
$SatO_2$	85.30±14.46	
**Outcome**		
Deaths	4	100%

*CRP: C-reactive protein (Normal value <0.5 mg/dL); **D-dimers (NV <0.5 μg/mL); #LDH: lactic dehydrogenase (Normal range: 120-246 U/L); &Ferritin (NR: 10-291 ng/mL).*
